# Unveiling the tumor microenvironment in colorectal cancer therapeutic resistance

**DOI:** 10.3389/fcell.2025.1753180

**Published:** 2026-02-02

**Authors:** Jiyu Han, Weitian Liang, Kai Li

**Affiliations:** 1 Editorial Department of “Progress of Anatomical Sciences”, China Medical University Journal Center, Shenyang, China; 2 Department of Intensive Care Medicine, The First Affiliated Hospital of China Medical University, Shenyang, China; 3 Department of Oncology Surgery, The First Affiliated Hospital of China Medical University, Shenyang, China

**Keywords:** chemotherapy, colorectal cancer, drug resistance, immunotherapy, targeted therapy, TIME, TME

## Abstract

Therapeutic resistance remains a major barrier to effective treatment in colorectal cancer (CRC), where the tumor microenvironment (TME) plays a pivotal role in modulating responses to chemotherapy, immunotherapy, and targeted therapies. This review synthesizes current evidence on how cellular and non-cellular TME components contribute to resistance mechanisms in CRC. Key immune cells, including T cells, macrophages, neutrophils, natural killer cells, dendritic cells, and myeloid-derived suppressor cells, orchestrate immunosuppressive networks that impair drug efficacy. For instance, regulatory T cells and M2-polarized macrophages promote chemoresistance via cytokine secretion and metabolic reprogramming, while neutrophils and myeloid-derived suppressor cells hinder immune checkpoint blockade through extracellular trap formation and T-cell exhaustion. Non-cellular elements, such as extracellular matrix remodeling, hypoxia-induced metabolic shifts, and dysregulated cytokines like IL-6 and TGF-β, further exacerbate resistance by fostering epithelial-mesenchymal transition and angiogenesis. Tables highlight specific molecular axes and therapeutic implications. By elucidating these interactions, this article underscores the potential of TME-targeted strategies, such as macrophage reprogramming, cytokine inhibition, and combination therapies, to overcome resistance and improve clinical outcomes in CRC patients. Future research should prioritize integrating TME biomarkers for personalized treatment approaches.

## Graphical Abstract

**Figure:**
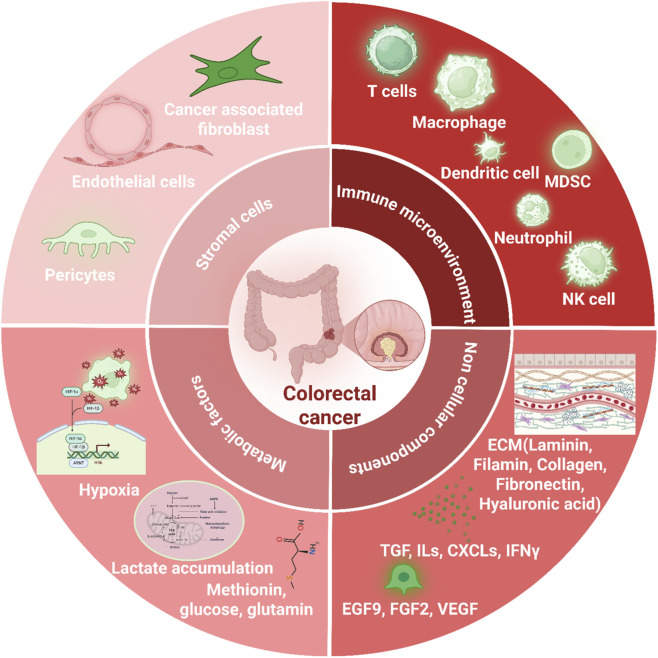

## 1 Introduction

Colorectal cancer (CRC) remains a major global health concern, ranking as the second leading cause of cancer-related mortality worldwide (Bray et al., 2024). According to the American Cancer Society, approximately 152,810 new CRC cases and 53,010 CRC-related deaths were estimated in 2024, illustrating its substantial clinical and societal burden (Siegel et al., 2024). Despite notable advances in early screening, surgical resection, and systemic therapies, outcomes for patients with advanced disease remain poor. Prognosis is strongly stage-dependent, with 5-year survival rates exceeding 90% for stage I patients, yet dropping to just 18.9% for those with stage IV disease (Hong et al., 2020). This stark disparity highlights the urgent need for improved prevention, early detection strategies, and more effective therapeutic interventions to enhance patient survival and quality of life (Zhang G. et al., 2024).

Therapeutic resistance has emerged as a critical obstacle in CRC management, undermining the success of conventional chemotherapy, targeted therapies, and immunotherapies. Drug resistance can be intrinsic, present at diagnosis, or acquired during treatment, often leading to tumor recurrence and metastasis (Haynes and Manogaran, 2025; Oktavianda and Giselvania, 2024). Mechanistically, resistance arises through diverse molecular and cellular pathways, including alterations in drug metabolism, efflux pump overexpression, epithelial-mesenchymal transition (EMT), cancer stem cell enrichment, DNA damage repair activation, and epigenetic reprogramming (Su et al., 2025; Gope et al., 2025; Zhou et al., 2024; Ebrahimnezhad et al., 2024). For example, resistance to 5-fluorouracil (5-FU) and oxaliplatin, cornerstones of CRC treatment, is frequently associated with dysregulation of apoptosis pathways and upregulation of multidrug resistance transporters (Ma et al., 2023). Similarly, mutations in KRAS, NRAS, and BRAF genes contribute to resistance to epidermal growth factor receptor (EGFR) targeted therapies, while microsatellite-stable (MSS) tumors often exhibit immune evasion, rendering immune checkpoint blockade (ICB) ineffective (Zhou et al., 2021; Lu et al., 2023). These challenges emphasize the urgent need for novel strategies to predict, prevent, and overcome drug resistance in order to improve clinical outcomes and achieve durable therapeutic responses.

Growing body of research highlights the tumor microenvironment (TME) as a pivotal contributor to CRC therapeutic resistance. The TME comprises a dynamic network of cellular components including immune cells, cancer-associated fibroblasts (CAFs), endothelial cells, and myeloid-derived suppressor cells (MDSCs), and non-cellular elements such as extracellular matrix (ECM) proteins, cytokines, chemokines, and metabolites (Xiao et al., 2025; Wu T. et al., 2025). This intricate ecosystem can be broadly classified into the tumor immune microenvironment (TIME), which governs immune surveillance and evasion, and the stromal microenvironment, which modulates structural and biochemical support for tumor growth (Bai et al., 2021; Wang Z-B. et al., 2024). In CRC, immunosuppressive immune cells such as regulatory T cells (Tregs), M2-polarized macrophages, and neutrophils impede cytotoxic T-cell responses, reducing the efficacy of ICB therapies (Zhu et al., 2022; Yu et al., 2025; Zhang et al., 2021). Meanwhile, hypoxia-induced signaling and ECM remodeling promote EMT and cancer stemness, contributing to chemoresistance and metastatic potential (Yuan et al., 2022). CAFs further exacerbate treatment resistance by secreting growth factors and exosomes carrying microRNAs that alter tumor signaling pathways (Wang J. et al., 2025). These findings underscore the need for therapeutic approaches that not only target tumor intrinsic mechanisms but also reprogram the TME to restore treatment sensitivity and enhance patient outcomes.

This review provides a comprehensive overview of the TIME’s role in CRC therapeutic resistance, focusing on the cellular and non-cellular components that mediate immune evasion and treatment failure. By dissecting the molecular and immunological pathways that underlie drug resistance, we aim to highlight promising therapeutic targets and emerging strategies to reprogram the TIME. Understanding these mechanisms is crucial for developing innovative combination approaches that enhance therapy sensitivity and improve survival outcomes for CRC patients.

## 2 Cellular components

### 2.1 Tumor immune microenvironments

#### 2.1.1 T cells

T cells, including CD4^+^ helper subsets and CD8^+^ cytotoxic T lymphocytes (CTLs), are central regulators of therapeutic response in CRC. Their functional state, ranging from effective cytotoxicity to exhaustion or suppression, critically determines sensitivity to chemotherapy, immunotherapy, and targeted agents.

In chemotherapy, resistance to 5-FU and oxaliplatin is closely linked to T-cell exclusion and immunosuppressive remodeling of the tumor immune microenvironment (TIME). CRC-derived CCL20, regulated through the FOXO1/CEBPB/NF-κB axis, recruits regulatory T cells (Tregs), thereby dampening CD8^+^ T-cell–mediated cytotoxicity and promoting 5-FU resistance (Wang et al., 2019). Conversely, IL-33, a multifunctional cytokine, enhances 5-FU sensitivity by activating CD4^+^ and CD8^+^ T cells through annexin A1-mediated signaling cascades. IL-33 triggers Th1 and Th2 responses in CD4^+^ T cells and effector-memory responses in CD8^+^ T cells, leading to tumor cell apoptosis and immune-mediated killing. This creates a positive feedback loop where injured CRC cells release IL-33, CXCL10, and CXCL13, further amplifying T cell responses. Notably, patients with high IL-33 and CD8^+^ T cell infiltration exhibit improved survival, underscoring the protective role of T cells in overcoming 5-FU resistance. However, IL-33’s dual role complicates its therapeutic potential, as it can also promote tumor cell proliferation in an autocrine manner, particularly in TME with low T cell infiltration (Song et al., 2023). Similarly, oxaliplatin efficacy is enhanced when DNA damage response pathways such as ATR are inhibited, leading to increased immunogenic signals and secondary activation of CD8^+^ T cells (Combès et al., 2019).

In immunotherapy, dysfunctional T-cell states are a defining feature of immune checkpoint blockade (ICB) resistance in microsatellite-stable (MSS) CRC. VEGF-A–driven induction of TOX enforces a transcriptional exhaustion program in T cells, limiting PD-1 blockade efficacy; dual inhibition of VEGF-A and PD-1 partially restores T-cell activity (Kim et al., 2019). The integrin αvβ6 further exacerbates ICB resistance by activating TGF-β signaling, which inhibits CD8^+^ T cell infiltration and cytotoxic activity. Unlike in triple-negative breast cancer, where TGF-β upregulates SOX4, αvβ6 in CRC directly suppresses T cells without SOX4 involvement. Blocking αvβ6 enhances T cell activation locally within the tumor, offering a promising strategy to overcome ICB resistance in MSS CRC (Busenhart et al., 2022). In parallel, RNA-modifying proteins such as YTHDF1 promote chemokine-driven recruitment of myeloid suppressor cells, indirectly reinforcing T-cell exhaustion and resistance to ICB (Bao et al., 2023).

Cetuximab, a monoclonal antibody targeting EGFR, is a key targeted therapy for CRC, but resistance limits its efficacy (Wahoski and Singh, 2024). Targeted therapy resistance also intersects with T-cell dysfunction. Cetuximab efficacy depends partly on immune-mediated tumor clearance, which is compromised when PD-L1 expression suppresses CD8^+^ T-cell activity. The HCG18/miR-20b-5p/PD-L1 axis exemplifies this mechanism, linking immune evasion directly to resistance against EGFR inhibition (Xu Y-J. et al., 2021).

The intricate interplay between T cells and the TIME underscores their central role in CRC therapeutic resistance across chemotherapy, immunotherapy, and targeted therapies (Table 1). In summary, T cells sit at the center of therapeutic resistance in CRC, where their dysfunction, driven by exhaustion, exclusion, or active suppression, underpins multiple shared resistance phenotypes. Mechanisms such as Treg-mediated inhibition, MDSC-induced exhaustion, and macrophage-secreted cytokines converge to impair CD8^+^ T-cell effector function, fostering immune exclusion and chronic immunosuppression. These processes are further amplified by non-cellular cues like hypoxia and TGF-β, which reinforce T-cell anergy across chemotherapy, immunotherapy, and targeted therapy contexts. Therapeutically, restoring T-cell primacy through combined blockade of exhaustion checkpoints, suppressive cytokines, or recruitment signals offers a rational strategy to dismantle interconnected immunosuppressive networks orchestrated by other TIME components.

**TABLE 1:** T cell-mediated mechanisms of drug resistance in colorectal cancer.

Upstream regulator	TIME	CRC and immune cell lines or tissu	Type of study	Therapeutics	Axis	Key finding	References
miR-146a	↑Treg, ↓CD4+ and CD8+ T cells	HT-29, HEK-293T	In vitro	5-fluorouracil (antimetabolite), irinotecan (topoisomerase I inhibitor)	TGF-β/IL-10	Boosts CD8+ function, anti-PD1 synergy	Khorrami et al. (2017)
Exosomal miR-208b	↑Tregs, ↓CD4+ and CD8+ T cells	SW480, SW480-OXA, NCM460, CT26	In vitro and in vivo	Oxaliplatin (alkylating agents)	miR-208b/PDCD4	Glycolysis promotes Treg escape	Ning et al. (2021)
PD-L1, β-catenin	↓CD8+ T cells, ↑Stromal PD-L1+ immune cells, ↑Nuclear β-catenin + tumor budding	human CRC and LAd-RC tissues	In vivo	Neoadjuvant chemoradiotherapy (NCRT), potential anti-PD-1/PD-L1	PD-L1/PD-1, β-catenin/CSC niche	Induces ICD, checkpoint synergy	Takahashi et al. (2022)
Lysophosphatidylcholine acyltransferase 2 (LPCAT2)	↑LD, ↓CD8+ T cells	SW620, HT29, CT26	In vitro and in vivo	5-fluorouracil (antimetabolite), Oxaliplatin (alkylating agents)	LPCAT2/lipid droplet (LD)/endoplasmic reticulum (ER) stress- calreticulin	Adaptive PD-L1 via CD8+	Cotte et al. (2018)
5-fluorouracil, Oxaliplatin	↑CD8+ T cells, ↓terminally exhausted T cells	MC38-CEA2, CT26	In vitro and in vivo	anti-PD1, 5-fluorouracil (antimetabolite), Oxaliplatin (alkylating agents)	CD8 T cell-mediated immunity	Lactic acid reduces CD8+	Guan et al. (2020)
circQSOX1	↑Treg, ↓CD8+ T cells	SW620, HCT116, LOVO, HT-29, CT26, MC38	In vitro and in vivo	anti-CTLA-4	miR-326/miR-330–5p/PGAM1	Kynurenine induces exhaustion	Liu et al. (2022a)
Oxaliplatin	↑CD8+ T cells, ↑CD4+ T cells	CT26, MC38, HCT-116, DLD-1	In vitro and in vivo	Anti-PD1, Anti-CTLA-4	PD-1/PD-L1, CTLA-4	Enhances T cell infiltration	Wang et al. (2017a)
​	↑CD8+ T cells, PD-L1+	​	In vitro and in vivo	anti-PD1/PD-L1	JAK/STAT1/IRF1, NF-κB	Overcomes KRAS resistance	Sudoyo et al. (2019)
Mutant KRAS	↓CD8+ T cells	MC38, MC38K, CRC PDX	In vitro and in vivo	Anti-PD1, Adoptive T-cell therapy	Lactic acid-NF-κB	Neutrophils promote EMT	Liu et al. (2023)
AT-rich interactive domain 5A (ARID5A)	↑CD8+ T cell exhaustion	HCT116, HT29	In vitro and in vivo	CAR-T cell therapy	ARID5A/IDO1/AhR	Boosts CD8+ function, anti-PD1 synergy	Wu et al. (2024)
CXCL10	↑CD8+ T, ↑CD4+ T, ↓Microvessel density	HT29, RKO, HCT116, SW480, LS174T, SW620, DLD1, CT26	In vitro and in vivo	Cetuximab (anti-EGFR mAb), anti-PD1	CXCL10-VCAN	Glycolysis promotes Treg escape	Yan et al. (2023)
CD16158V-Chimeric Receptor (CR)	↑CD8+ T cells, ↑IFNγ, ↑TNFα	HCT116, 293T	In vitro and in vivo	Cetuximab (anti-EGFR mAb)	CD16-CR signaling	Induces ICD, checkpoint synergy	Arriga et al. (2020)
Stromal cell-derived factor-1 (CXCL12/SDF-1)	↑CD15high neutrophils, GZMKhigh CD8+ effector memory cells	Caco-2, HT-29 MTX, HMEC-1, MC38	In vitro and in vivo	​	SDF-1-CXCR4/CXCR2	Adaptive PD-L1 via CD8+	Tiberti et al. (2022)

#### 2.1.2 Macrophages

Tumor-associated macrophages (TAMs) are highly plastic immune cells that critically shape therapeutic resistance in CRC. M2-polarized TAMs dominate resistant tumors, where they integrate cytokine signaling, metabolic reprogramming, and immune suppression across chemotherapy, immunotherapy, and targeted therapy contexts (Wang S. et al., 2024).

Macrophages play a pivotal role in mediating resistance to 5-FU, a cornerstone chemotherapeutic agent in CRC (Gharzeddine, 2022). In chemotherapy resistance, TAMs attenuate the cytotoxic effects of 5-FU through metabolic and paracrine mechanisms. Polyamine production via ornithine decarboxylase suppresses apoptosis in CRC cells by inhibiting JNK/caspase-3 signaling (Zhang et al., 2016). Another key resistance axis involves macrophage-secreted interleukin-6 (IL-6). In co-culture and *in vivo* models, IL-6 released by TAMs activates the IL6R/STAT3 pathway in CRC cells, subsequently downregulating the tumor-suppressive microRNA miR-204-5p. This axis not only reduces drug-induced apoptosis but also facilitates sustained chemoresistance. Furthermore, decreased levels of miR-155-5p in TAMs lead to upregulation of the transcription factor C/EBPβ, which enhances IL-6 transcription and reinforces this chemoresistance loop (Yin et al., 2017). Chemokines including CCL22 further promote EMT and PI3K/AKT signaling, linking macrophage infiltration to both chemoresistance and tumor invasiveness (Wei et al., 2019).

Despite the success of ICB in some cancers, its efficacy in CRC, especially MSS subtypes, remains limited, largely due to the immunosuppressive actions of TAMs. These macrophages, particularly those polarized to the M2 phenotype, inhibit T cell infiltration and activation, fostering a “cold” tumor immune environment unresponsive to PD-1/PD-L1 inhibitors (Cai et al., 2024). Regulators such as GPSM1 and CDA enhance TAM recruitment and polarization, reinforcing immune exclusion and resistance to PD-1 blockade (Chen et al., 2025a; Scolaro et al., 2024). Furthermore, IDO1 expression in tumor cells contributes to macrophage-mediated immune evasion. IDO1 induces kynurenine production, suppresses T cell function, and promotes M2 polarization via the IL6/JAK2/STAT3 pathway. Inhibition of IDO1 not only alters macrophage behavior but also reprograms the TIME to favor T cell-mediated anti-tumor immunity, enhancing the effectiveness of PD-1 blockade in MSS CRC (Guangzhao et al., 2025).

In targeted therapy, TAMs also undermine responses to EGFR inhibition. lncRNA-driven M2 polarization, exemplified by the HCG18/miR-365a-3p/FOXO1/CSF-1 axis, correlates with cetuximab resistance and poor clinical outcomes (Gao et al., 2022). Additionally, Macrophage migration inhibitory factor (MIF) has been identified as a driver of cetuximab resistance. Overexpression of MIF in CRC cells promotes mitogen-activated protein kinase (MAPK) and AKT pathway activation, supporting cell survival despite EGFR inhibition. MIF’s interaction with CXCR4 and CD74 on tumor and immune cells underpins its resistance-inducing activity. Importantly, dual targeting of MIF and EGFR—using MIF inhibitors like 4-IPP or ISO-1 in combination with cetuximab—synergistically enhances apoptosis and inhibits downstream survival signaling, overcoming resistance (Russo et al., 2019).

Across chemotherapy, immunotherapy, and targeted therapy modalities, macrophages, particularly M2-polarized TAMs, serve as central architects of colorectal cancer drug resistance (Table 2). Macrophages, particularly the M2-polarized subset, emerge as versatile orchestrators of resistance, linking cellular and non-cellular compartments through cytokine secretion, metabolic reprogramming, and physical remodeling of the stroma. Their pro-tumorigenic activities overlap extensively with those of MDSCs and CAFs, collectively sustaining immune suppression, metabolic rewiring, lactate and polyamine production, and stromal conditioning that shields tumor cells from drug-induced stress. By recruiting Tregs and excluding cytotoxic T cells, M2 macrophages reinforce immune exclusion phenotypes. Targeting macrophage polarization or key effector molecules therefore represents a high-leverage intervention capable of disrupting multiple convergent resistance modules simultaneously.

**TABLE 2:** Decoding macrophage-dependent drug resistance in colorectal cancer.

Upstream regulator	TIME	Cell lines	Typ of study	Therapeutic type	Axis	Highlightes	References
CCL17, CCL22	↑TAMs (M2-polarized macrophages)	DLD1, SW480, SW620, THP-1, hPBMC	*In vitro* and *in vivo*	5-Fluorouracil (antimetabolite), HA15 (GRP78 inhibitor)	CCL17/CCL22-CCR4-PI3K/AKT-IP3R-ATF6-GRP78-MRP1	TAM-driven GRP78-MRP1 chemoresistance	Zhang et al. (2023a)
Hypoxia-induced HIF2α	↑ dihydropyrimidine dehydrogenase (DPD) in TAMs	RAW264.7, CT-26, RKO, HT-29, human colorectal primary and secondary tumors	*In vitro* and *in vivo*	5-fluorouracil (antimetabolite)	HIF2α/DPD	HIF2α-induced DPD inactivates 5-FU	Jang et al. (2018)
TGFβ, CSF1	↑Tregs, ↓CD8+ T cells, ↑CSF1R + TAMs, ↑PD-L1+	SW480, SW620, HCT116, HCT15, HT29, LoVoWT, LoVoOxR, CT26, THP-1	*In vitro* and *in* *vivo*	Oxaliplatin (alkylating agent), 5-fluorouracil (antimetabolite), folinic acid, TGFβR inhibitor (galunisertib)	TGFβ/Smad2/PD-L1, CSF1-CSF1R/TAM	CSF1-recruited TAMs upregulate PD-L1	Chen et al. (2022a)
lncRNA MIR155HG, ANXA2	↑M2 macrophages, ↓M1 macrophages	Caco2, HT29, SW480, HCT116, LoVo, HCoEpiC, THP-1, human CRC tissues	*In vitro* and *in vivo*	Oxaliplatin (alkylating agent)	MIR155HG/miR-650/ANXA2	MIR155HG stabilizes ANXA2, promotes M2	Zhou et al. (2022)
Macrophage migration inhibitory factor(MIF)	↓Macrophages	mouse xenograft tumor model	*In vitro* and *in vivo*	Oxaliplatin (alkylating agent)	MIF/CXCR7/CTCF	MIF upregulates CXCR7, enhances resistance	Hu et al. (2024a)
Bromodomain-containing protein 4 (BRD4)	↓M2-like TAMs, enhanced anti-tumor microenvironment	HCT116, HT29, Brd4-CKO mouse models	*In vitro* and *in vivo*	Oxaliplatin (alkylating agent)	BRD4/Serpine1/PAI-1/SMAD	BRD4 drives PAI-1, supports M2 polarization	Cheon et al. (2018)
Fibroblast Activation Protein (FAP)	↑M0 and M2 macrophages, ↓CD8+ T cells, ↓CD4+ memory resting T cells	LS174T, HCT8, HCT116, BALB/c-nu mice	*In vitro* and *in vivo*	5-Fluorouracil (antimetabolite), Irinotecan (CPT-11, topoisomerase I inhibitor), Doxorubicin	FAP/MPRIP/RhoA/Hippo/YAP	FAP activates RhoA/YAP, recruits M2 TAMs	Jang et al. (2018)
METTL3, TRAF5	↑M2-polarized TAMs, ↑MDSC, ↓CD4+ and CD8^+^ T cells	HCT116, HT29 CRC tissues (Oxaliplatin -resistant and oxaliplatin -sensitive patients)	*In vitro* and *in* *vivo*	Oxaliplatin (alkylating agent), anti-PD1	METTL3/TRAF5/m6A modification, BHLHE41-CXCL1/CXCR2	METTL3 in M2 TAMs drives oxaliplatin resistance	Lan et al. (2021)
Adenosine Deaminase Acting on RNA 1 (ADAR1)	↑M2 macrophages (CD68^+^, CD163+), ↓M1 macrophages	HCT116, HT29, THP-1, human CRC tissue	*In vitro* and *in vivo*	Oxaliplatin (alkylating agent), Filgotinib (JAK inhibitor)	ADAR1/AZIN1/GLI1/SPP1/NFκB	ADAR1 edits GLI1, promotes M2 polarization	Umeda et al. (2025)
​	↑M2 macrophages, ↑PD-L1+ M2 macrophages	Human mCRC tissues	*In vivo*	Cetuximab (anti-EGFR mAb)	​	Post-treatment increase in PD-L1+ M2 TAMs	Kim et al. (2022)
MIF	↓Macrophages	HCT116, LOVO, SNUC1, Colo201, Colo205, LS174T, HT29, SNU81, SNU175	*In vitro*	Refametinib (MEK inhibitor), 4-IPP (MIF inhibitor)	MIF/STAT3/MAPK	MIF mediates MEK inhibitor resistance	Cheon et al. (2018)
CXCL10	↑M1 macrophages	HT29, RKO, HCT116, SW480, LS174T, SW620, DLD1, CT26	*In vitro*	Cetuximab (anti-EGFR mAb), anti-PD1	CXCL10-VCAN	CXCL10 enhances cetuximab/anti-PD1 response	Cheon et al. (2018)
Mutant KRAS, MIF	↓Macrophages	KRAS MT (HCT116, DLD1), KRAS WT (Caco-2, HT-29), patient-derived CRC tissues	*In* *vitro* and *in vivo*	Cetuximab (anti-EGFR mAb)	MIF/AKT/NF-κB	KRAS-induced MIF causes cetuximab resistance	Jang et al. (2018)
miR-1226-5p	↑M2 macrophages	HCT116, SW480, THP-1, CRC patient-derived organoids, CRC patient tissues	*In vitro* and *in vivo*	Radiotherapy	circSLC43A1/miR-1226-5p/IRF1/TGF-β	miR-1226-5p induces M2, promotes radioresistance	Cheon et al. (2018)

#### 2.1.3 Neutrophils

Neutrophils play a pivotal role in mediating resistance to chemotherapy in CRC, particularly with capecitabine treatment. Research has identified that the expression level of CD16 on neutrophils in peripheral blood is significantly downregulated in capecitabine-resistant CRC patients (Shibata et al., 2023). This reduction in CD16 expression is associated with adverse clinical outcomes and appears earlier than detectable changes on CT scans, suggesting its potential as an early prognostic marker. RNA sequencing further revealed that CD16low/- neutrophils in resistant patients exhibit lower expression of neutrophil-related genes compared to CD16^+^ neutrophils in capecitabine-sensitive patients, indicating that these cells may represent an immature neutrophil population. Notably, CD16 expression on neutrophils positively correlates with the abundance of anti-tumor immune cell subsets, such as CD8^+^ T cells, CD4^+^ T cells, NK cells, and monocytes, highlighting neutrophils’ influence on the broader immune landscape in chemotherapy resistance (Lu et al., 2020).

Transitioning to immunotherapy resistance, neutrophils significantly contribute to the ineffectiveness of immune checkpoint inhibitors, particularly in MSS or mismatch repair-proficient (pMMR) CRCs, which constitute approximately 85% of cases. In preclinical models, neutrophil extracellular traps (NETs) have been shown to form in the livers of mice with colorectal liver metastasis (CRCLM), promoting resistance to anti-PD-1 blockade (Zhang H. et al., 2024). The administration of DNase I, which degrades NETs, significantly reduces tumor-associated neutrophils and NET formation, thereby enhancing CD8^+^ T cell infiltration and cytotoxicity, reversing resistance to anti-PD-1 therapy in both microsatellite instability-high (MSI-H) and MSS CRCLM models (Zhang et al., 2021). Furthermore, in mismatch repair-deficient (MMRD) tumor models, tumor-infiltrating neutrophils impair the response to anti-PD-1 therapy, particularly in the 4T1 breast cancer model, where high TIN abundance correlates with resistance. Combining anti-PD-1 with anti-CTLA-4 or anti-CD25 antibodies to deplete Tregs or limit TIN accumulation restores immunotherapy efficacy, underscoring neutrophils’ role in fostering an immunosuppressive TME (Nebot-Bral et al., 2022). The neutrophil-to-lymphocyte ratio (NLR) has also emerged as a dynamic biomarker, with changes in NLR within the first 2 months of treatment predicting anti-PD-L1 response in MMRD tumors, offering a non-invasive tool for early therapeutic adjustment.

In the context of targeted therapies, such as anti-vascular endothelial growth factor (anti-VEGF) treatment, neutrophils are critical mediators of resistance, particularly in CRCLM with a replacement histopathological growth pattern (HGP). Studies have demonstrated that lysyl oxidase-like 4 (LOXL4) is transcriptionally upregulated in neutrophils within the TME of replacement HGP CRCLM, compared to desmoplastic HGP tumors or adjacent normal liver tissue. This LOXL4 expression, inducible by lipopolysaccharide and TNF-α, is higher in circulating neutrophils of cancer patients than in healthy controls, positioning LOXL4-expressing neutrophils as a potential biomarker for identifying resistant CRCLM subtypes (Palmieri et al., 2020). Additionally, in mouse models of CRC with chemically induced colitis, elevated levels of granulocyte colony-stimulating factor (G-CSF) and its target, Bv8/PROK2, expressed by tumor-infiltrating neutrophils, promote resistance to anti-VEGF therapy. Combining antibodies targeting G-CSF or Bv8/PROK2 with anti-VEGF treatment suppresses tumor progression and myeloid cell infiltration, highlighting neutrophils’ contribution to an inflammatory TME that undermines targeted therapy efficacy (Itatani et al., 2020).

Neutrophils contribute to therapeutic resistance primarily through inflammatory and immunosuppressive mechanisms that intersect with broader TIME networks. NET formation, immature neutrophil expansion, and cytokine-driven recruitment create barriers to T-cell infiltration and sustain chronic inflammation, aligning with immune exclusion and stromal conditioning phenotypes observed across other myeloid populations. Their interplay with macrophages and MDSCs amplifies systemic immunosuppression, while interactions with endothelial cells promote angiogenesis and anti-VEGF resistance. Disrupting neutrophil trafficking, NETosis, or G-CSF/LOXL4 signaling could thus simultaneously alleviate multiple resistance nodes, enhancing the efficacy of both cytotoxic and immunotherapeutic regimens.

#### 2.1.4 Natural killer (NK) cells

NK cells play a pivotal role in modulating the TIME, particularly in overcoming therapeutic resistance in CRC. Recent research has elucidated their significant impact on oxaliplatin-resistant CRC cell lines, such as OR-DLD1 and OR-RKO, demonstrating that co-culturing these cells with NK cells significantly inhibits their growth both *in vitro* and *in vivo* using mouse xenograft models. This growth suppression is mediated through a novel regulatory cascade involving the downregulation of WBSCR22, a gene previously implicated in oxaliplatin resistance. Specifically, NK cell co-culture upregulates miR-146b-5p, a microRNA that directly targets WBSCR22 mRNA, leading to its reduced expression. The critical role of miR-146b-5p is underscored by experiments showing that its specific inhibitor attenuates NK cell-induced growth repression, highlighting NK cells as key orchestrators in reversing oxaliplatin resistance through this molecular pathway (Zhao et al., 2018). The interplay between NK cells and tumor metabolism further enriches our understanding of their role in CRC resistance. A systematic analysis of multiomics and survival data has revealed a novel association between mitochondrial acetyl-CoA acetyltransferase 1 (ACAT1) and NK cell infiltration, which influences CRC progression. Under immune stimulation, ACAT1 is phosphorylated at serine 60, translocates to the nucleus, and acetylates lysine 146 of p50 (*NF-κB*1), enhancing the expression of immune-related factors that promote NK cell recruitment and activation. However, in nutrient-poor tumor microenvironments, this process is hindered, correlating with reduced NK cell infiltration and poorer prognosis. These findings emphasize NK cells’ dependency on metabolic cues within the tumor microenvironment to exert their antitumor effects, offering a potential avenue for enhancing NK cell-based therapies (Wei et al., 2025). Moreover, NK cells exhibit heightened efficacy against dMMR CRC, which is characterized by high immune cell infiltration and MHC Class I defects. Studies using CRISPR-Cas9-generated MLH1, DR4, and DR5 knockout cell lines, along with NK92-MI or murine NK cells, have shown that dMMR CRC cells are more sensitive to NK cell-mediated cytotoxicity compared to pMMR cells. This sensitivity is mediated by upregulated death receptors (DR4/5), which facilitate interleukin-12 secretion, sustaining NK cell viability in dMMR CRC. *In vivo* studies further confirm that NK cell depletion promotes dMMR CRC tumor growth, while NK cell transfer inhibits lung metastasis in models expressing DR4/5, with TP53 enhancing DR4/DR5 expression (Yang et al., 2024). These results collectively position NK cells as critical mediators in targeting dMMR CRC, offering a promising strategy to overcome therapeutic resistance.

Beyond traditional bulk-omics approaches, single-cell technologies are revolutionizing our understanding of TME heterogeneity and its contribution to therapeutic resistance. By resolving cellular states and intercellular networks at unprecedented resolution, single-cell RNA sequencing and related methods have identified critical immune subsets that dictate response to immune checkpoint inhibitors in other solid tumors (Guan and Quek, 2025). For example, in hepatocellular carcinoma, single-cell analyses combined with immunophenotyping revealed distinct natural killer cell populations associated with ICI responsiveness, enabling immune subtyping and prediction of docetaxel synergy (Lai et al., 2025). Applying these methodologies to CRC, particularly microsatellite-stable tumors with inherently “cold” microenvironments, holds immense promise for uncovering resistance-driving cell–cell interactions, defining therapy-responsive cellular niches, and guiding the development of personalized TME-modulating strategies.

**FIGURE 1:**
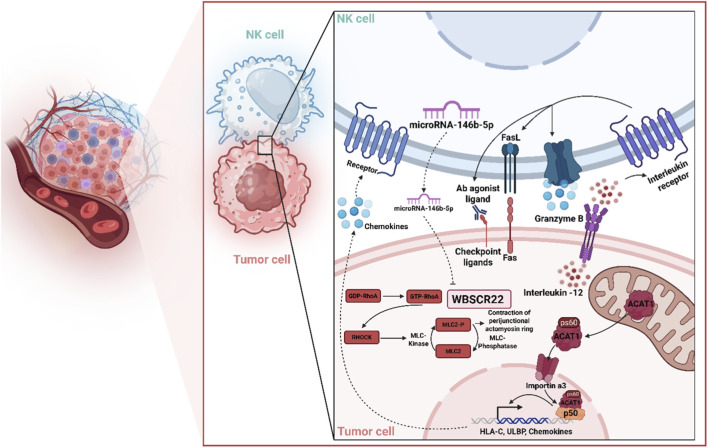
Mechanistic insights into NK cell-mediated regulation of CRC drug resistance. NK cells modulate the tumor immune microenvironment (TIME) and enhance therapeutic responses in CRC by targeting multiple resistance-associated pathways. NK cell co-culture elevates miR-146b-5p levels, leading to suppression of WBSCR22, a key factor in oxaliplatin resistance, thereby reducing tumor growth. The RHO-ROCK/MLCK-MLC2 axis is implicated in cell-in-cell (CIC) structure formation and NK cell resistance, with its inhibition restoring NK cytotoxicity. Additionally, immune stimulation triggers ACAT1 phosphorylation and nuclear translocation, enhancing NK cell recruitment through transcriptional activation of immune-related genes. dMMR CRC demonstrates heightened vulnerability to NK cytotoxicity via DR4/DR5 upregulation and interleukin-12 secretion, supporting NK cell viability and antitumor activity. Collectively, NK cells orchestrate antitumor responses by rewiring resistance mechanisms, metabolism, and death receptor signaling, offering a foundation for NK-targeted immunotherapies in drug-resistant CRC.

Together, these findings underscore the multifaceted role of NK cells in counteracting colorectal cancer drug resistance. As illustrated in Figure 1, by downregulating resistance-associated genes like WBSCR22 via miR-146b-5p, leveraging metabolic pathways through ACAT1-mediated immune activation, and exploiting DR4/5-mediated cytotoxicity in dMMR CRC, NK cells emerge as central players in reshaping the tumor immune microenvironment. These insights pave the way for developing targeted NK cell-based immunotherapies to enhance treatment outcomes in oxaliplatin-resistant and dMMR CRC, addressing a critical unmet need in clinical oncology. Although often suppressed within the CRC TIME, NK cells retain potent capacity to counteract resistance when appropriately activated. Their ability to target stem-like and metabolically altered tumor cells bridges innate and adaptive immunity, countering metabolic rewiring and immune exclusion driven by myeloid suppressors and fibroblasts. Enhanced NK activity, via death receptor upregulation or metabolic rescue, can disrupt the protective niches maintained by MDSCs and M2 macrophages, indirectly restoring CD8^+^ T-cell function. Integrating NK-cell–directed approaches with therapies that relieve myeloid suppression therefore holds promise for overcoming multifactorial resistance phenotypes.

#### 2.1.5 Dendritic cells (DCs)

DCs play a pivotal role in shaping the immune response within the CRC tumor microenvironment, significantly influencing therapeutic resistance. The immunosuppressive TME in CRC, driven by factors such as thymidine phosphorylase (TYMP) overexpression, promotes resistance to immunotherapies and adoptive cell therapies (ACTs) by inhibiting DC effector functions. TYMP-induced secretion of IL-10 and TGF-β in the TME suppresses DC activity, leading to increased CD8^+^ T-cell exhaustion and the conversion of effector T cells into Tregs, fostering an immunosuppressive environment that hampers immunotherapy efficacy. This dynamic underscores DCs as critical mediators in counteracting or exacerbating CRC resistance, depending on therapeutic modulation (Paladhi et al., 2022).

DCs are central to bridging innate and adaptive immunity, and their dysfunction contributes to poor immunotherapy outcomes (Singer et al., 2025). TYMP inhibition with tipiracil hydrochloride (TPI) induces endoplasmic reticulum stress, releasing damage-associated molecular patterns (DAMPs) like calreticulin, HMGB1, and ATP, priming immunogenic cell death (ICD). At 150 mg/kg/day, TPI suppresses TYMP and induces ICD in preclinical CRC models but damages the host immune system, limiting prognostic benefits. A suboptimal TPI dose reduces CRT exposure, increases PD-L1 expression, yet enhances effector TILs like CTLs and NK cells while decreasing exhausted CD8^+^ T cells, Tregs, and TAMs. This highlights DCs’ role in balancing immunostimulation and immunosuppression, as DAMPs promote DC maturation and T-cell-mediated antitumor activity. To counter chemotherapy-induced immunosuppression, TPI combined with TLR7 agonists like imiquimod in CT26 CRC mouse models enhances DC activation, increasing cytokine secretion and costimulatory molecule expression, CD40, CD70, and CD86. This primes NK and T cells, induces ICD, recruits T cells, depletes TAMs and Tregs, and transforms Tregs into Th1 effector phenotypes, enhancing tumor cell killing via TCR-MHC interactions. Type I interferon signatures amplify these effects, reinforcing DCs’ role in robust antitumor immunity (Paladhi et al., 2022). DCs loaded with tumor cell lysates or CSC-enriched antigens further enhance antitumor responses by presenting tumor antigens via MHC class I and II molecules (Del Prete et al., 2023). In CRC, lysate-pulsed DCs stimulate antigen-specific CD4^+^ and CD8^+^ T-cell responses, countering tumor escape mechanisms. However, immunosuppressive molecules like CTLA-4, expressed on DCs, reduce their maturation and antigen-presenting capacity by upregulating IL-10 and diminishing IL-12 production. Silencing CTLA-4 in CRC cell lysate-loaded DCs via siRNA transfection significantly enhances CD11c, CD40, and CD86 expression, boosting TNF-α and IL-12 secretion while reducing IL-10. This modification increases CD3^+^ T-cell proliferation and IFN-γ and IL-4 production, indicating a shift toward Th1 and Th2 responses. Surprisingly, FOXP3 expression, a Treg marker, increases in these co-cultures, suggesting a complex regulatory dynamic. These findings position DCs as key players in overcoming immunosuppressive barriers when modified to enhance stimulatory capacity (Ghorbaninezhad et al., 2022).

DCs also mediate responses to novel agents like F1929-1458, an NF-κB pathway agonist that induces tumor cell stress and DAMP release, activating cGAS-STING and NLRP3 inflammasome pathways in DCs. This leads to type I IFN and IL-1β production, enhancing DC maturation and antigen presentation, which drives T-cell-mediated immunity. In CSC-enriched colorectal tumor models, low-dose F1929-1458 remodels the TME, increasing T-cell infiltration and reducing immunosuppression, demonstrating DCs’ role in translating innate signals into adaptive immunity. Similarly, chemotherapy agents like SN-38, an irinotecan metabolite, upregulate MHC class I expression via TAP1 and TAP2 pathways, enhancing DC antigen presentation, though increased PD-1/PD-L1 expression may counteract these benefits, necessitating combination strategies (Chen et al., 2025b). In CSC-focused therapies, DCs loaded with chemo-resistant CSC-like cells (CD44+/CD24−) from cisplatin-treated EC cell lines exhibit mature DC phenotypes (high CD11c, CD83, CD86 expression) and induce robust antitumor responses in SEC-bearing mice. Combining CSC-loaded DC vaccines with low-dose cisplatin significantly inhibits tumor growth, increases IFN-γ production, and upregulates p53 expression, which promotes tumor cell apoptosis. These outcomes highlight DCs’ ability to target resistant CSC populations, enhancing chemotherapy efficacy and prolonging survival. However, limitations such as the use of heterogeneous CSC-like populations and undefined CSC antigens underscore the need for further research (El-Ashmawy et al., 2019).

Dysfunctional dendritic cells represent a critical upstream bottleneck in antitumor immunity, perpetuating immune exclusion and tolerance across the TIME. Their impaired maturation and antigen presentation, exacerbated by TYMP, TGF-β, and suppressive cytokines from macrophages and MDSCs, prevent effective priming of T and NK cells, allowing chronic immunosuppression to dominate. Conversely, strategies that restore DC function, TLR agonism, TYMP inhibition, or *ex vivo* loading, can initiate a positive feedback loop, enhancing effector infiltration and countering stromal conditioning. Positioning DCs as central nodes for therapeutic reprogramming offers a unifying approach to break interconnected resistance circuits.

#### 2.1.6 MDSC

MDSCs have emerged as key players in shaping the immunosuppressive TME and mediating resistance to therapies in CRC. These cells, broadly categorized into polymorphonuclear (PMN-MDSCs) and monocytic subsets (M-MDSCs), accumulate significantly in CRC patients, especially at advanced stages, and contribute to both disease progression and poor response to treatment (Zeng et al., 2024).

Accumulating evidence indicates that MDSCs are actively involved in resistance to conventional chemotherapeutic agents. In particular, resistance to oxaliplatin, a widely used first-line chemotherapeutic in CRC, has become a major clinical hurdle (Wang et al., 2022). While the cytotoxic effect of oxaliplatin is well-characterized, its underlying immunological implications have only recently begun to be elucidated. MDSCs, along with their differentiated progeny such as TAMs, have been implicated in attenuating chemotherapeutic efficacy. Emerging research has revealed that oxaliplatin resistance in CRC is associated with the immunosuppressive activity of MDSCs and their differentiation into TAMs. In tumor-bearing mice, oxaliplatin treatment led to a reduction of M-MDSCs and M1-type TAMs at the tumor site, indicating that insufficient activation of M1 macrophages may undermine oxaliplatin efficacy. Combining oxaliplatin with immunostimulatory agents, such as TLR7/8 agonists like R848, successfully reprogrammed MDSCs toward a pro-inflammatory M1-like phenotype, enhancing both apoptosis in cancer cells and inhibition of migration and invasion. This approach demonstrated that targeting MDSC differentiation and function can overcome oxaliplatin resistance, highlighting the potential of immunologic adjuvants as enhancers of chemotherapy response in resistant CRC (Liu et al., 2020). In clinical contexts, the combination of oxaliplatin with fluorouracil and folinic acid (FOLFOX) has demonstrated selective depletion of MDSCs, particularly in patients with initially elevated PMN-MDSC levels. This subset of patients also showed improved T cell infiltration, suggesting that chemotherapeutic-induced MDSC suppression may enhance antitumor immunity. Mechanistically, oxaliplatin has been found to impair the immunosuppressive functions of MDSCs by downregulating ARG1 and NOX2 and by inhibiting NF-κB activation. Such findings position MDSC-targeted strategies as a valuable adjunct to chemotherapy, potentially overcoming resistance in selected CRC patients (Gunarsa et al., 2025). Further reinforcing this concept, 5-FU exerts its antitumor activity not only through direct cytotoxicity but also by modulating the immune microenvironment. Specifically, 5-FU activates p53 signaling in MDSCs, leading to upregulation of Fas and promoting FasL-induced apoptosis. The result is a marked reduction in MDSC populations and an increase in CTL infiltration in both murine models and human CRC patients. Importantly, this immunomodulatory mechanism remains effective even in cases where tumor cells harbor TP53 mutations, as MDSCs typically retain wild-type TP53 function. These findings underscore the dual action of chemotherapy—direct tumoricidal effects and immunologic reprogramming of the TME (Yang et al., 2023).

In the realm of immunotherapy, MDSCs are now recognized as significant contributors to resistance against ICB (Li et al., 2021). In most MSS CRC cases, ICB therapies such as PD-1/PD-L1 inhibitors show limited efficacy. This has been attributed in part to MDSC-mediated suppression of CTLs via both PD-L1–dependent and–independent mechanisms. High levels of MDSCs in the tumor correlate with poor infiltration of CD8^+^ T cells and impaired activation of antitumor immunity. Recent studies using organoid models and *in vivo* mouse systems have demonstrated that the effectiveness of PD-1 blockade is associated with reduced MDSC frequencies and functionality. Activated T cells can induce apoptosis in MDSCs through IFN-α/β and TNF-α signaling, primarily via the TRAIL–TRAILR interaction, though the involvement of other pathways like Fas/FasL remains context-dependent. These findings reveal a complex crosstalk between T cells and MDSCs that governs the success of immunotherapy. Furthermore, the selective targeting of MDSCs, via cytokine modulation or combination therapies—has been shown to augment the therapeutic efficacy of ICB in otherwise unresponsive CRC cases (Chen et al., 2021a).

On a molecular level, MDSC accumulation in CRC has been linked to epigenetic regulation through the RNA methyltransferase METTL3. This enzyme enhances the expression of CXCL1 via m6A methylation of the transcription factor BHLHE41, promoting the recruitment of MDSCs through the CXCL1/CXCR2 axis. METTL3-deficient CRC models exhibit diminished MDSC infiltration and improved CD8^+^ T cell activation, thereby suppressing tumor growth. Notably, the combination of METTL3 inhibition with PD-1 blockade has shown synergistic effects, pointing to METTL3 as a viable therapeutic target for overcoming immunotherapy resistance (Chen H. et al., 2022).

Taken together, MDSCs represent a central immunological barrier in CRC treatment resistance, affecting both chemotherapy and immunotherapy outcomes (Table 3). Emerging strategies that reprogram MDSCs, either by reversing their differentiation, inducing apoptosis, or blocking their recruitment, offer promising avenues to enhance therapeutic efficacy. In particular, combining oxaliplatin with agents that activate M1-like macrophages may overcome immunologically driven chemoresistance. Targeting MDSCs, through cytotoxic agents like 5-FU, immunomodulators such as TLR agonists, or epigenetic inhibitors like those against METTL3, offers a promising avenue to enhance therapeutic efficacy. These strategies hold the potential to reprogram the tumor immune microenvironment, restore effective antitumor immunity, and ultimately improve clinical outcomes in CRC patients.

**TABLE 3:** MDSC-associated mechanisms contributing to therapeutic resistance in CRC.

Upstream regulator	TIME	Cell lines	Typ of study	Therapeutic type	Axis	Highlightes	References
SLC2A3, POU2F2	↑NK cells (low NKRS), ↓NK cells (high NKRS)	SW480, RKO, TCGA-COAD, GSE39582	*In vitro*	Anti-PD-1, Anti-PD-L1, Anti-CTLA-4	TGF-β signaling pathway	NKRS predicts poor prognosis; better ICB response in low NKRS	Li et al. (2023a)
CT45A1	↑NK cell resistance, ↑Homotypic CIC structure formation	DLD-1, HCT-15, SW480, HT29, NK-92MI, human PB NK cells, MSI-H CRC tissues	*In vitro* and *in vivo*	Anti-PD-L1	RHO-ROCK/MLCK-MLC2	Enhances NK resistance and CIC shielding	Teng et al. (2025)
Sphingosine Kinase 2 (SphK2)	↑MDSC, ↓CD4+ and CD8^+^ T cells	HCT116, C57BL/6J mice (WT), SphK2 Tg mice	*In vitro* and *in vivo*	5-Fluorouracil (antimetabolite)	IL-6/STAT3/ARG-1	Promotes immunosuppression and 5-FU resistance	Li et al. (2024a)
SLC25A22	↓MDSC, ↑CD8+ T cells, ↑CD4+ T cells	CT26, MC38, DLD1, APC-KRAS mutant organoids, PBMC humanized mice	*In vitro* and *in vivo*	Anti-PD1	SLC25A22/asparagine /SRC/ETS2/CXCL1-CXCR2	Reduces MDSC recruitment; synergizes with anti-PD1	Zhou et al. (2023)
Methyltransferase like 3 (METTL3)	↑MDSC, ↓CD4^+^ and CD8^+^ T cells	CT26 and MC38, 293T cell	*In vitro* and *in vivo*	Anti-PD1	BHLHE41-CXCL1/CXCR2	Enhances MDSC migration; synergizes with anti-PD1	Chen et al. (2022b)
Death Receptor 5 (DR5)	↑CD8+ T cells, ↓MDSCs	MC38	*In vitro* and *in vivo*	Anti-PD-L1	DR5/TRAIL, PD-1/PD-L1	Depletes MDSCs; enhances anti-PD-L1 efficacy	Tang et al. (2022)
USP15	↑MDSC, ↓CD8+ T cells	CT26, CMT93, HT29, HCT116, HCT15, SW48, SW480, SW620, SW948, DLD1, RKO CRC and metastatic tumor models of human and mouse	*In vitro* and *in vivo*	Anti-PD-1	USP15/SMYD3/CCL2	Stabilizes SMYD3; promotes MDSC recruitment	Han et al. (2025)

The cellular components of the CRC tumor immune microenvironment function not as isolated players but as an interconnected network that amplifies therapeutic resistance through several shared phenotypes: immune exclusion, profound T-cell suppression and exhaustion, metabolic competition, and stromal reinforcement. Recurrent signaling hubs, such as TGF-β, IL-6/STAT3, hypoxia-driven HIF-1α, and chemokine axes like CXCL12/CXCR4 and CXCL1/CXCR2, emerge across T cells, macrophages, neutrophils, NK cells, dendritic cells, and MDSCs, creating self-reinforcing immunosuppressive loops. Disrupting these convergent nodes, CSF1R inhibitors for macrophage reprogramming, CXCR2 antagonists for myeloid trafficking, or multi-cytokine blockade, offers high-leverage opportunities for rational combination strategies that could restore sensitivity to chemotherapy, immune checkpoint blockade, and targeted therapies across molecular subtypes of CRC.

### 2.2 Stromal cells

#### 2.2.1 Cancer-associated fibroblasts

Through intricate cellular communication, particularly via exosome-mediated mechanisms, CAFs enhance tumor progression, metastasis, and drug resistance, making them critical targets for improving CRC treatment outcomes (Kamali Zonouzi et al., 2022).

CAFs play a dominant role in driving chemotherapy resistance in CRC, particularly against first-line treatments such as fluoropyrimidine-based and platinum-based chemotherapies (Wang J. et al., 2025). This resistance is largely mediated through exosome-driven cellular communication, which promotes aggressive tumor phenotypes and undermines therapeutic efficacy. Specifically, CAFs secrete exosomes enriched with miR-92a-3p, a microRNA that targets FBXW7 and MOAP1 in CRC cells. FBXW7, also known as Hcdc4, is a tumor suppressor that inhibits cancer progression by suppressing mTOR signaling, while MOAP1 promotes apoptosis via BAX activation. By downregulating these targets, miR-92a-3p enhances cell stemness, EMT, and inhibits apoptosis, leading to increased metastasis and resistance to 5-FU and oxaliplatin. This is evidenced by elevated levels of stemness markers CD133 and CD44, which increase the proportion of CSCs with self-renewal and chemoresistance properties. Furthermore, CAFs promote the dedifferentiation of non-CSCs into CSC-like phenotypes, amplifying the resistant cell population (Hu et al., 2019). In addition, CAFs-derived exosomes deliver lncRNA CCAL, which interacts with the mRNA-stabilizing protein HuR to upregulate β-catenin mRNA and protein levels, activating the β-catenin pathway. This pathway is critical for CSC maintenance and oxaliplatin resistance, as it enhances cell survival and proliferation under chemotherapeutic stress (Deng et al., 2020). Similarly, exosomal circular RNA cricN4BP2L2, by binding to EIF4A3, activates the PI3K/AKT/mTOR signaling axis, further promoting CSC characteristics and oxaliplatin resistance in CRC cells, such as the LoVo cell line. *In vitro* and *in vivo* experiments demonstrate that knocking down cricN4BP2L2 in CAFs-derived exosomes significantly reduces these resistance traits, underscoring its mechanistic role (Qu et al., 2022). Moreover, CAFs from chemoresistant patients (R-CAFs) exhibit distinct functional differences compared to those from chemosensitive patients, as co-culture experiments reveal that R-CAF-derived exosomes enhance CRC cell proliferation, suppress apoptosis, and confer resistance to cisplatin (Shi et al., 2023). Beyond exosomal mechanisms, CAFs contribute to chemotherapy resistance through soluble factors in conditioned media. Both untreated and chemotherapy-treated CAFs (exposed to 5-FU, Oxa, or DMSO) secrete soluble factors that enhance CSC sphere-forming capacity, a hallmark of chemoresistance, in CRC cell lines and xenografts. This phenomenon, termed soluble factor-mediated drug resistance (SFM-DR), is evident in experiments using fibroblast cell lines and primary CAFs, where conditioned media promote CSC enrichment and resistance to chemotherapy-induced cell death. Notably, CD133+ CRC cells, identified as putative CSCs, show inherent resistance to chemotherapy, with their proportion increasing post-treatment, further highlighting CAFs’ role in priming CSCs. The TOP-GFP experimental system, which monitors Wnt signaling activity, confirms that CAFs enhance CSC drug resistance through paracrine signaling (Hu et al., 2015; Sun et al., 2025). Collectively, these findings emphasize CAFs’ multifaceted role in fostering chemotherapy resistance through exosomal miRNAs, lncRNAs, circular RNAs, soluble factors, and ECM remodeling, all converging to enhance CSC phenotypes and suppress apoptosis in CRC.

In the realm of immunotherapy, particularly ICB, CAFs expressing high levels of fibroblast activation protein (FAP) are instrumental in fostering an immunosuppressive TME, leading to anti-PD-1 resistance in CRC. High FAP-expressing CAFs increase MDSCs while reducing T-cell infiltration and activation, creating an immunosuppressive phenotype associated with poor survival and immunotherapy failure. Mechanistically, these CAFs secrete CCL2, which recruits MDSCs and regulatory T cells, further exacerbating immunosuppression. Experimental models, such as the CT26 syngeneic tumor, demonstrate that FAP-high CAFs induce anti-PD-1 resistance, which can be reversed by targeting FAP with inhibitors like FAPi, suggesting a potential combinatorial strategy with ICB. This highlights CAFs’ critical role in modulating the immune landscape to undermine immunotherapy efficacy in CRC (Chen et al., 2017).

CAFs also contribute to resistance against targeted therapies, such as cetuximab (anti-EGFR) and anti-VEGF treatments, by remodeling the ECM and promoting angiogenesis. Matrices derived from CAFs, particularly those expressing SNAI1, confer greater resistance to oxaliplatin and cetuximab compared to normal fibroblast-derived matrices, with SNAI1 regulating drug resistance and metabolism gene expression in CRC cells (Galindo-Pumariño et al., 2022). Furthermore, CAFs expressing high levels of sulfatase 1 (SULF1) enhance VEGFA bioavailability by modifying heparan sulfate proteoglycans, promoting ECM deposition and angiogenesis, which supports tumor progression and resistance to anti-VEGF therapies. CAFs-derived exosomes containing VEGFA directly regulate CRC cell viability, apoptosis, and angiogenesis, further contributing to resistance against anti-VEGF treatments. *In vivo* studies confirm that CAFs from chemoresistant patients deliver VEGFA via exosomes, accelerating CRC progression. These findings emphasize CAFs’ role in orchestrating ECM remodeling and angiogenic signaling, which drives resistance to targeted therapies in CRC (Wang H. et al., 2024).

The interplay between CAFs and CRC cells via exosomes, soluble factors, and ECM remodeling creates a multifaceted resistance network across chemotherapy, immunotherapy, and targeted therapies. Exosomal miR-92a-3p, highly expressed in the serum of metastatic CRC patients, serves as a potential biomarker for predicting metastasis and therapy resistance. Similarly, lncRNA CCAL and circular RNA cricN4BP2L2 highlight the molecular complexity of CAF-mediated resistance, while FAP and SULF1 expression in CAFs offers prognostic insights and therapeutic targets (Table 4). The consistent role of CAFs in promoting CSC phenotypes, immunosuppression, and angiogenesis underscores their central contribution to therapeutic resistance in CRC, necessitating novel strategies targeting CAFs to enhance treatment efficacy and improve patient outcomes.

**TABLE 4:** CAF contributions to colorectal cancer drug resistance.

Stromal cell component	Role of stromal cell components	Target	Cell lines	Typ of study	Therapeutic type	Axis	Highlightes	References
CAFs	Oncogene	lncRNA H19	HCT116 and SW480 mouse xenografts	*In vitro* and *in vivo*	Oxaliplatin (alkylating agent)	H19/β-catenin/miR-141	Exosomal H19 promotes stemness and resistance	Ren et al. (2018)
CAFs	Oncogene	Soluble factors (cytokines, chemokines, growth factors)	HT29, DLD-1, HCT116, HTOXAR3	*In vitro*	, 5-fluorouracil (antimetabolite), Oxaliplatin (alkylating agents)	PI3KCA/AKT, JAK/STAT, P38, CHK2, Survivin	Soluble factors enhance DNA repair and survival	Gonçalves-Ribeiro et al. (2016)
CAFs	Oncogene	TIAM1	HCT116, SW480, SW620, DLD-1, HCT15, CaCO2, COLO320, HT29, RKO Mice xenografts	*In vitro* and *in vivo*	5-fluorouracil (antimetabolite), Oxaliplatin (alkylating agent), irinotecan (topoisomerase I inhibitor)	Wnt/TIAM1/Rac1	TIAM1 drives stemness and chemoresistance	Izumi et al. (2019)
CAFs	Oncogene	CXCL12	LoVo, HCT116, THP-1	*In vitro* and *in vivo*	Cisplatin (alkylating agent)	CXCL12/M2 macrophage polarization	CXCL12 induces M2 polarization and resistance	Jiang et al. (2023)
CAFs	Oncogene	Soluble factors (TGF-β1, FGF, IL-6, HGF, OPN, SDF1)	HCT116, HCT15, SW480	*In vitro* and *in* *vivo*	KRAS-targeted inhibition (siRNA)	KRAS, TGF-β, NOTCH, WNT, MYC, EMT, Hippo	Factors bypass KRAS inhibition via stemness	Oliveira et al. (2024)
CAFs	Oncogene	IL-1α	LS411N, HT29, SW480, SW837 and SW1463	*In vitro* and *in vivo*	5-fluorouracil (antimetabolite), Oxaliplatin (alkylating agents)	IL-1/NF-κB/p38	IL-1 promotes senescence and resistance	Nicolas et al. (2022)
CAFs	Oncogene	Platinum (oxaliplatin)	HT29-M6, PDO, CCD-18Co, CAF1, CAF2	*In vitro*, *in vivo*	Oxaliplatin (alkylating agent)	TGF-beta/IL11/POSTN	Retained oxaliplatin upregulates IL11/POSTN	Linares et al. (2023)
CAFs	Oncogene	SFRP1	SW480, CT26	*In vitro* and *in vivo*	Echinomycin (HIF1 inhibitor)	SFRP1/FGFR2/HIF1	SFRP1 drives metastasis via HIF1 axis	Liu et al. (2025a)
CAFs	Oncogene	IFNα/β signaling, MHC class II	Patient-derived organoids	*In vitro*	5-Fluorouracil (antimetabolite), Oxaliplatin (alkylating agent)	JAK/STAT	Modulates immune response and resistance	Ryu et al. (2024)
CAFs	Oncogene	LINC00355	Not specified	*In vitro* and *in vivo*	Oxaliplatin (alkylating agent)	LINC00355/miR-34b-5p/CRKL	Exosomal LINC00355 induces EMT and resistance	Hu et al. (2024b)
CAFs	Oncogene	Fibroblast-related gene signature (FRGS)	HCT116, SW480, HT29, DLD-1, HTOXAR3	*In vitro*, *in vivo*, retrospective cohort analysis	Oxaliplatin (alkylating agent), 5-fluorouracil (antimetabolite), PD-1 inhibitors	Immune response, IL-6, CCL2, TNF-α, IFN-α, IFN-γ, IL-2	FRGS predicts prognosis and immune suppression	Chen et al. (2021b)
CAFs	Oncogene	TGF-β2, GLI2, HIF-1α	TS1, TS2, TS3, CT34, CT128	*In vitro* and *in vivo*	5-fluorouracil (antimetabolite), Oxaliplatin (alkylating agent)	HIF-1α/TGF-β2/GLI2	Hypoxia and TGF-β2 activate GLI2 in stem cells	Tang et al. (2018)
CAFs	Oncogene	RBCK1	SW480, SW620, HT-29, CACO2, LOVO	*In vitro* and *in vivo*	5-Fluorouracil (antimetabolite), Oxaliplatin (alkylating agent)	Nanog, Oct4, Sox2, Klf4	RBCK1 enhances stemness and chemoresistance	Liu et al. (2019)

#### 2.2.2 Endothelial cells

Endothelial cells, encompassing microvascular and macrovascular types, interact dynamically with cancer cells, modulating tumor progression and drug response through angiogenic and signaling pathways. Understanding these interactions provides critical insights into overcoming resistance mechanisms in CRC treatment.

Endothelial cells significantly influence the response of CRC cells to chemotherapeutic agents like 5-FU. Studies have shown that the supernatant from naive CRC cell lines, such as CCL228 and its metastatic derivative CCL227, exerts a modest stimulatory effect on primary human dermal microvascular endothelial cells (HDMEC) proliferation after 2 days, while having little impact on human umbilical vein endothelial cells (HUVECs) (Wang R. et al., 2017). However, after 4 days, this effect reverses, inhibiting HDMEC growth and even suppressing HUVEC proliferation. This suggests a time-dependent shift in the tumor-endothelial interaction, potentially due to the accumulation of inhibitory factors in the tumor supernatant. Notably, only naive cell lines CCL228 and CCL227 induce this stimulatory effect, highlighting a differential behavior compared to chemoresistant subclones. The secretion of VEGF by these cells further complicates this dynamic. While CCL227 secretes significantly higher VEGF level compared to CCL228 after 4 days, resistant subclones produce less VEGF, inversely correlating with their degree of chemoresistance. However, these VEGF concentrations remain below the 10 ng/mL threshold required for endothelial cell stimulation *in vitro*, indicating that VEGF alone is insufficient to drive angiogenesis in this context. Moreover, the growth stimulatory effect of VEGF on HDMEC is matrix-dependent, with vitronectin or Tenascin-C coatings enhancing stimulation, unlike collagen I or uncoated surfaces. This underscores the critical role of the extracellular matrix in modulating endothelial cell responses to chemotherapeutic resistance (Schönau et al., 2007). The interaction between CRC cells and endothelial cells also extends to three-dimensional models, which mimic metastatic processes. Research demonstrates that lymphatic endothelial cell (LEC) barriers are more sensitive to tumor-induced clear cell invasion domain (CCID) formation than blood endothelial cell (BEC) barriers, particularly in the presence of 5-FU-resistant CRC cells. Aggressive 5-FU-resistant CCL227-RH cells enhance LEC mobility compared to naive CRC cells, facilitating tumor breaching through lymphatic vasculature. The miR200 family members, miR200c, miR141, and miR429, inhibit CCID formation in both CRC/LEC and CRC/BEC models by targeting ZEB2 and SNAI, key mediators of EMT and metastasis. However, miR200a and miR200b unexpectedly induce CCID formation, suggesting complex regulatory mechanisms. Interestingly, treatments like mocetinostat and sulforaphane, which influence histone acetylation, attenuate BEC barrier disintegration independently of miR200 expression, indicating alternative pathways in maintaining endothelial integrity. These findings highlight that endothelial cells not only respond to chemotherapeutic stress but also actively contribute to the metastatic potential of resistant CRC cells, linking chemotherapeutic resistance to vascular breaching (Holzner et al., 2016).

Targeted therapies, such as anti-EGFR and anti-VEGF agents, also face resistance modulated by endothelial cell interactions. Long-term treatment with EGFR inhibitors like cetuximab (C225) or gefitinib (ZD1839) in GEO colon cancer xenografts leads to the emergence of resistant cell lines with elevated VEGF expression and activated phospho-mitogen-activated protein kinase (MAPK). These resistant cells exhibit a 5–10-fold increase in cyclooxygenase-2 and VEGF, suggesting that endothelial cells may be indirectly activated through enhanced angiogenic signaling. However, treatment with ZD6474, a dual inhibitor of VEGF receptor-2 and EGFR, effectively blocks tumor growth in these resistant xenografts, indicating that targeting VEGF signaling can overcome EGFR inhibitor resistance (Ciardiello et al., 2004). This is further supported by studies on gamma-synuclein (SNCG), which is overexpressed in tumor vasculature and promotes resistance to bevacizumab, an anti-VEGF monoclonal antibody. SNCG activates VEGFR2, creating a feedback loop with bevacizumab that exacerbates resistance. Combining Bev with an anti-SNCG antibody significantly reduces tumor growth and metastasis in bevacizumab-resistant HT29 xenografts by inhibiting SNCG-VEGFR2 signaling, underscoring the critical role of endothelial cells in mediating resistance to anti-VEGF therapies (liu et al., 2024).

Additionally, VEGF’s autocrine and paracrine roles in CRC cells influence their response to targeted therapies. Homozygous deletion of VEGF alleles in CRC cells results in decreased cell growth, increased apoptosis, and heightened sensitivity to 5-FU, mediated by upregulation of proapoptotic mediators, caspase-3, cleaved PARP, Bax, and downregulation of survivin. This suggests that VEGF, beyond its angiogenic role, supports CRC cell survival, impacting both chemotherapeutic and targeted therapy outcomes (Samuel et al., 2011). Regorafenib, a multi-kinase inhibitor targeting VEGF-R, fibroblast growth factor receptor (FGF-R), and PDGF-R, paradoxically induces malignant phenotypes in CRC cells by blocking VEGF signaling, enhancing apoptosis resistance and migration. This effect is specific to VEGF-R inhibition, as selective inhibitors of FGF-R or PDGF-R do not replicate these phenotypes. These findings emphasize that endothelial cells, through their interaction with VEGF and other signaling pathways, are central to the resistance mechanisms against targeted therapies, necessitating strategies that address both tumor and endothelial compartments (Tomida et al., 2017).

As indicated in Table 5, the interplay between endothelial cells and CRC cells reveals a multifaceted role in therapeutic resistance. In chemotherapeutic contexts, endothelial cells modulate tumor cell behavior through dynamic responses to tumor-derived factors like VEGF, with resistant subclones exhibiting reduced angiogenic potential yet enhanced metastatic capabilities via EMT and vascular breaching. In targeted therapies, endothelial cells contribute to resistance by amplifying SNCG or MAPK angiogenic signaling or responding to VEGF-mediated survival signals in tumor cells. The differential behavior of microvascular and macrovascular endothelial cells, coupled with matrix-dependent effects and miRNA regulation, underscores their complexity in CRC resistance. Therapeutic strategies, such as combining anti-VEGF agents with anti-SNCG antibodies or using multi-kinase inhibitors like ZD6474, highlight the potential to target endothelial cell interactions to overcome resistance. These insights pave the way for integrated approaches that address the tumor-endothelial axis to enhance the efficacy of both chemotherapeutic and targeted therapies in CRC.

**TABLE 5:** Endothelial cell contributions to colorectal cancer drug resistance.

Stromal cell component or their growth factor	Role of stromal cell components	Cell lines	Typ of study	Therapeutic type	Axis	Highlightes	References
VEGFA	Oncogene	SW480, HCT116 mouse xenografts, GEMM	*In vitro* and *in vivo*	Bevacizumab (anti-angiogenic antibody)	BMAL1/REVERBA/VEGFA	BMAL1 drives bevacizumab resistance via VEGFA	Burgermeister et al. (2019)
Microvascular endothelial (mvE) cells	Tumor suppressor	TCGA, GSE39582, GSE28702 cohorts	In silico	5-Fluorouracil (antimetabolite), Oxaliplatin (alkylating agent)	VEGF, S1P-related genes, EMT, myogenesis	mvE abundance predicts better chemotherapy response	Oshi et al. (2021)
mvE	Tumor suppressor	CT-26, SW-480, SW-620, HUVECs	*In vitro*, *in vivo*	Metformin, Cyclophosphamide (alkylating agent), 5-Fluorouracil (antimetabolite)	Caspase signaling, endothelial apoptosis	Metformin overcomes chemoresistance via vascular maturity	Li et al. (2023b)
Immunomodulatory endothelial cells (IMECs)	Tumor suppressor	Not specified	In silico	Bevacizumab (anti-angiogenic antibody), Immunotherapy anti-PD-1	MHC-II, VEGF	IMECs link angiogenesis and MHC-II antigen presentation	Wen (2024)
VEGF	Oncogene	Not specified	Cohort	5-Fluorouracil (antimetabolite)	VEGF/SPF/TS	High VEGF/SPF predicts early recurrence	Cascinu et al. (2001)

#### 2.2.3 Pericytes

Pericytes, critical components of the TME in CRC, significantly influence resistance to both immunotherapy and targeted therapies. By stabilizing tumor vasculature and modulating immune responses, pericytes play a pivotal role in hindering the efficacy of treatments like chemotherapy and antiangiogenic therapies, while also offering potential avenues for overcoming resistance when strategically targeted (Jordan et al., 2025). This section explores their dual role, weaving together their impact on immunotherapy and targeted therapies for enhanced clarity and flow.

In immunotherapy, pericytes contribute to an immunosuppressive TME that restricts immune cell infiltration, particularly in MSS CRC and the CMS4 subtype. Overexpression of olfactomedin-like 3 (OLFML3) correlates with increased pericyte coverage of tumor blood vessels, stabilizing them and limiting access for T, B, and NK cells. This is exacerbated by VEGF-A, which downregulates adhesion molecules like VCAM-1 and ICAM-1, impeding T-cell adhesion and extravasation. Additionally, tumor-associated endothelial cells express PD-1, further suppressing T-cell activation. Pericytes thus form a vascular barrier that shields tumors from immune attack, reinforcing resistance to immunotherapy. Targeting pericytes offers a breakthrough. Anti-OLFML3 antibodies disrupt pericyte-endothelial interactions, reducing tumor angiogenesis, lymphangiogenesis, and TAMs while boosting NK-like T-cell recruitment. This shift fosters an immunosupportive TME, enhancing immunotherapy outcomes. Combining anti-OLFML3 with anti-PD-1 checkpoint inhibitors amplifies infiltration by B cells, T lymphocytes, and NKT cells, significantly improving tumor suppression compared to monotherapy (Stalin et al., 2021). Similarly, endostatin (Endostar) normalizes tumor vasculature by increasing pericyte coverage, as marked by elevated α-smooth muscle actin (α-SMA) expression. This facilitates greater CD8^+^ T-cell infiltration and interferon-gamma (IFN-γ) secretion, correlating with improved survival in patients treated with Endostar and PD-L1 inhibitors. By modulating pericyte function, these therapies transform the TME, enhancing immune cell access and immunotherapy efficacy (Chu et al., 2022).

In targeted therapies, particularly antiangiogenic treatments like bevacizumab, pericytes drive resistance by maintaining vascular stability (Besler et al., 2022). Bevacizumab targets endothelial cells but leaves pericyte coverage intact, allowing rapid vessel regrowth post-treatment through compensatory proangiogenic responses, such as elevated VEGF family member expression. This contributes to therapeutic failure, as pericytes and their associated vascular basal membranes enable tumors to evade antiangiogenic effects. In CRCLM, vessel co-option, a non-angiogenesis-dependent process, further complicates resistance. Hepatic stellate cells (HSCs), acting as liver-specific pericytes, facilitate tumor cell hijacking of pre-existing sinusoidal blood vessels, promoting an immunosuppressive TME. Bevacizumab induces fibroblast activation protein-alpha (FAPα) expression in HSCs, driving EMT and MDSC recruitment, which inhibits CD8^+^ T-cell infiltration. Targeting pericytes can counteract this resistance. The FAPα-activated prodrug Z-GP-DAVLBH disrupts co-opted vessels by targeting FAPα+ HSCs, offering a novel strategy to overcome antiangiogenic resistance. Pericytes’ role in stabilizing tumor vasculature thus fuels resistance, but their targeted disruption or modulation can enhance the efficacy of antiangiogenic therapies (Qi et al., 2022).

Pericytes are central to CRC therapeutic resistance, acting as gatekeepers of the TME by stabilizing tumor vasculature and limiting immune cell infiltration in immunotherapy, while enabling vessel co-option and rapid vessel regrowth in targeted therapies. Therapies like anti-OLFML3 and Z-GP-DAVLBH disrupt pericyte-driven resistance, dismantling vascular and immunosuppressive barriers. Meanwhile, endostatin’s vascular normalization leverages pericytes to enhance drug and immune cell delivery, though its transient effects highlight the need for combination strategies. By targeting pericytes’ dual role in resistance and therapeutic potential, novel treatments can transform the TME, paving the way for more effective CRC therapies.

## 3 Non-cellular components

### 3.1 ECM

The ECM profoundly influences drug resistance in CRC, shaping the effectiveness of both chemotherapy and targeted therapies. As tumors advance, ECM remodeling, driven by genetic mutations and changes in the tumor microenvironment, fosters resistance through mechanisms such as EMT, activation of survival signaling pathways, and metabolic reprogramming. This dynamic interplay between tumor cells and the ECM creates a complex environment that hinders therapeutic success (He et al., 2022). By exploring the ECM’s pivotal role in resistance to treatments like 5-FU and anti-VEGF agents such as bevacizumab, researchers can uncover strategies to overcome these barriers and improve patient outcomes.

The ECM significantly drives resistance to chemotherapeutic agents like 5-FU, a mainstay of CRC treatment. Research using cultured cell-derived decellularized matrices, which replicate the native ECM of tumor tissues at varying malignancy stages, has shown that ECM remodeling enhances 5-FU resistance. Specifically, matrices from highly malignant HT-29 colorectal tumor cells increased 5-FU resistance compared to those from less malignant SW480 cells or normal CCD-841-CoN cells. This resistance stemmed from ECM-driven activation of the Akt pathway and upregulation of drug efflux transporters ABCB1 and ABCC1, without promoting cell proliferation. These findings underscore the ECM’s critical role in fostering a microenvironment that supports chemoresistance during tumor progression, positioning staged tumorigenesis-mimicking matrices as valuable tools for studying ECM-mediated resistance and screening anti-cancer drugs (Hoshiba and Tanaka, 2016). Moreover, ECM remodeling promotes chemoresistance by facilitating EMT, a process linked to heightened malignancy and drug resistance. *In vitro* ECM models mimicking colorectal tumor tissues at different malignancy levels revealed that high-malignancy matrices, enriched with CS chains, amplified TGF-β-induced EMT and ABCB1 upregulation in HT-29 cells. In contrast, matrices with reduced CS chains failed to support these changes, highlighting how specific ECM components drive EMT and chemoresistance. This suggests that ECM remodeling during tumor progression alters matrix composition to bolster resistance mechanisms (Hoshiba, 2018).

The ECM protein ECM1 further amplifies 5-FU resistance. CRC patients resistant to 5-FU showed elevated ECM1 expression, which correlated with poorer overall and disease-free survival. *In vitro* and *in vivo* studies using HCT15 and 5-FU-resistant HCT15/FU cell lines demonstrated that ECM1 regulates resistance via the PI3K/AKT/GSK3β signaling pathway, modulating apoptosis and EMT induction. Knocking down ECM1 reduced resistance, while its overexpression enhanced it, positioning ECM1 as a promising therapeutic target to reverse 5-FU resistance. These insights emphasize the ECM’s multifaceted role in chemoresistance, driven by matrix remodeling, specific components, and signaling pathways (Long et al., 2022). Additionally, the long non-coding RNA LINC01347 contributes to ECM-related chemoresistance. Elevated LINC01347 expression in advanced-stage CRC correlated with poor prognosis and 5-FU resistance. Mechanistically, LINC01347 sponges miR-328-5p, upregulating LOXL2, an ECM-modifying enzyme. This LINC01347/miR-328-5p/LOXL2 axis promotes cell proliferation and 5-FU resistance, with LOXL2 knockdown attenuating resistance. These findings highlight ECM remodeling mediated by LOXL2 as a key driver of chemoresistance, offering a potential biomarker and therapeutic target (Zheng et al., 2021). The ECM component laminin also influences chemosensitivity. Knockdown of the α5 laminin chain in CRC cells triggered Wnt- and mTORC1-dependent dedifferentiation and activated ER-stress signaling, enhancing 5-FU sensitivity. This demonstrates that specific ECM components can modulate chemotherapy responses by altering cellular differentiation, further illustrating the ECM’s complex role in chemoresistance (Maltseva et al., 2020).

The ECM also critically shapes resistance to targeted therapies, such as bevacizumab, an anti-VEGF monoclonal antibody used in advanced CRC (Saif, 2013). Analysis of liver metastases from CRC patients revealed that nonresponders to bevacizumab exhibited increased ECM deposition and activation of the fatty acid oxidation (FAO) pathway compared to responders. In mouse models of metastatic CRC, anti-VEGF therapy induced ECM remodeling, increasing matrix stiffness and FAO in tumor cells. This resistance was mediated by HSCs, which, via the FAK/YAP pathway, secreted free fatty acids that tumor cells absorbed as metabolic substrates, enhancing FAO. Inhibiting FAO with etomoxir or suppressing HSC lipolysis via FAK/YAP inhibition improved bevacizumab efficacy, underscoring the ECM’s role in driving metabolic cross-talk that fuels resistance to antiangiogenic therapy. Matrix stiffness, a key biophysical property of the ECM, activates lipolysis in HSCs, promoting FAO in tumor cells and creating a resistant tumor microenvironment. This ECM-mediated metabolic reprogramming highlights the ECM’s pivotal role in limiting bevacizumab efficacy and suggests that targeting these interactions could enhance therapeutic outcomes (Zheng et al., 2023).

The ECM’s influence on drug resistance in CRC spans both chemotherapy and targeted therapies, with overlapping mechanisms that enhance tumor resilience. ECM-driven EMT and signaling pathways, such as PI3K/AKT, contribute to both 5-FU and bevacizumab resistance, indicating shared molecular drivers. The upregulation of drug efflux transporters like ABCB1 in chemotherapy resistance parallels the metabolic adaptations driven by ECM stiffness in bevacizumab resistance, both facilitated by ECM remodeling. Specific ECM components, including CS chains, ECM1, and laminins, modulate resistance across therapies by altering tumor cell behavior and microenvironmental dynamics (Table 6). These interconnected mechanisms position the ECM as a central orchestrator of resistance, making it a critical target for therapeutic intervention.

**TABLE 6:** ECM components in CRC drug resistance.

Extracellular matrix and their component	Role of ECM	Regulator of ECM	Cell lines	Typ of study	Therapeutic type	Axis	Highlightes	References
ECM, hyaluronic acid (HA), and sulfated glycosaminoglycans (sGAGs)	Tumor suppressor	Bevacizumab	CT26, SL4	*In vitro*	Bevacizumab (anti-angiogenic antibody)	Anti-VEGF Therapy/hypoxia/HA/sGAG Upregulation	Anti-VEGF upregulates HA, causing resistance	Burgermeister et al. (2019)
Fibronectin (FN)	Oncogene	Integrin αvβ1	SW480, CT26	*In vitro* and *in vivo*	5-Fluorouracil (antimetabolite)	Integrin αvβ1/CDC42/YAP-1/SOX2	FN drives resistance via YAP-1/SOX2 activation	Ye et al. (2020)
FN	Oncogene	Integrin α5β1	HT-29, HT-29S	*In vitro*	Irinotecan (topoisomerase I inhibitor)	Integrin α5β1/PI3K/Akt	FN matrix activates PI3K/Akt in resistance	Berg (2015)
Collagen	Oncogene	DDR1	MC38	*In vitro* and *in* *vivo*	Irinotecan (topoisomerase I inhibitor)	DDR1/Collagen/MMP2/P-gp	DDR1-collagen barrier promotes drug efflux	Cui et al. (2024)
Extradomain-B fibronectin (EDB-FN)	Oncogene	ZD2-targeted contrast agent MT218	DLD-1, RKO, DLD1-DR, RKO-DR	*In vitro* and *in vivo*	MK2206-HCl (pan-AKT inhibitor)	EDB-FN/MRMI	Elevated EDB-FN marks drug-resistant CRC	Vaidya et al. (2020)
Filamin A (FLNA)	Oncogene	c-Met, smad2	HCT116, HT29	*In vitro* and *in vivo*	5-Fluorouracil (antimetabolite), Crizotinib (c-Met inhibitor), LY294002 (PI3K/AKT inhibitor), LY2109761 (smad2 inhibitor)	c-Met/AKT/FLNA/smad2/EMT	FLNA phosphorylation drives EMT-mediated resistance	Cheng et al. (2020)
Laminin, 67-kDa laminin receptor (67LR)	Oncogene	67LR	SW480	*In vitro*	Doxorubicin (anthracycline antibiotic)	67LR/Bax/Bcl-2	67LR inhibits apoptosis, promoting resistance	Lu et al. (2016)
Laminin subunit alpha 3 (LAMA3)	Oncogene	Hippo-YAP pathway	HCT116, HT29	*In vitro* and *in vivo*	Oxaliplatin (alkylating agent)	Hippo-YAP pathway	LAMA3 activates YAP, reducing oxaliplatin sensitivity	Li et al. (2025a)

### 3.2 Cytokines and chemokines

These signaling molecules influence tumor progression, immune evasion, and drug resistance by modulating drug efflux, immune suppression, and cell survival pathways. This section elucidates their roles across different therapeutic modalities, weaving together insights from chemotherapy, immunotherapy, and targeted therapies to highlight their interconnected impact on CRC resistance and potential therapeutic strategies (Table 7).

**TABLE 7:** Cytokine and chemokine contributions to colorectal cancer drug resistance.

Chemokines or cytokines	Role of cytokine or chemokine	Cell lines	Type of study	Therapeutic type	Axis	Highlightes	References
IL-17A	Oncogene	CT26, MC38, HT29, SW480, and SW620	*In vitro* and *in vivo*	anti-PD-1	P65/NRF1/miR-15b-5p	Promotes anti-PD-1 resistance via PD-L1 upregulation	Liu et al. (2021a)
IL-17	Oncogene	HCT116	*In vitro*	Cisplatin (alkylating agent)	p-Akt/Bax/Bcl-2/mTOR	Inhibits apoptosis, promotes cisplatin resistance	Sui et al. (2019)
IL-22	Oncogene	SW480, SW620, HCT116, Colo205, LoVo	*In vitro*	5-Fluorouracil (antimetabolite), Oxaliplatin (alkylating agent)	IL-22/STAT3/IL-8	Activates STAT3, induces chemoresistance	Wu et al. (2014)
IFNγ	Oncogene in low doses and tumor suppressor in high doses	HCT116, SW480, CT26	*In vitro* and *in vivo*	anti-PD-1	JAK-STAT	Induces PD-L1, dual role in anti-PD-1 response	Yuan et al. (2019)
IFNγ	Oncogene	HCT14, HCT19, HCT20, HCT23, HCT24	*In vitro* and *in vivo*	Trametinib, selumetinib (MEK inhibitors), gefitinib (EGFR inhibitor)	RAS/IFN/STAT	Confers MEK inhibitor resistance via STAT signaling	Sakahara et al. (2019)
IFNγ	Oncogene	CT26, DLD1	*In vitro* and *in vivo*	anti-PD-1	IFNγ/PD-L1	Enhances PD-L1, synergizes with anti-PD-1	Tang et al. (2024)
IFNγ	Oncogene	MC38	*In vitro* and *in vivo*	anti-PD-1	IFNGR1	IFNGR1 loss causes anti-PD-1 resistance	Nie et al. (2025)
CXCL3	Oncogene	iKAP, MC38	*In vitro* and *in vivo*	anti-PD-1	KRAS*/IRF2/CXCL3/CXCR2	Recruits MDSCs, drives anti-PD-1 resistance	Liao et al. (2019)
CXCL1/5	Oncogene	​	*In vitro*	Cetuximab (EGFR inhibitor)	SNAI2/NFKB/CXCR/MMPI/EGF	Mediates EMT and cetuximab resistance	Park et al. (2022)
CXCL-13	Oncogene	DLD-1, HCT116	*In vitro* and *in vivo*	5-Fluorouracil (antimetabolite)	CXCL-13/CXCR5	Autocrine loop promotes 5-FU resistance	Zhang et al. (2020a)
CXCL10/11	Tumor suppressor	HT29, HCT116, SW620, NCM460, MC38	*In vitro* and *in vivo*	anti-PD-1	RIG‐I/STAT1/CXCL10/11	Enhances T-cell infiltration via STAT1	Nie et al. (2025)
TGF-β	Oncogene	HCT-8, HCT-8/5-FU, SW480, SW480/5-FU	*In vitro*	5-Fluorouracil (antimetabolite)	TGF-β signaling pathway	Inhibits apoptosis, promotes 5-FU resistance	Zhu et al. (2020)
TGF-β	Oncogene	RKO, SW480, HCT116, SW620	*In vitro* and *in vivo*	Oxaliplatin (alkylating agent)	TGF-β/Smad	Promotes EMT and oxaliplatin resistance	Gao et al. (2023)
TGF-β	Oncogene	HCT-8, HCT-8/5-FU, SW480, SW480/5-FU	*In vitro*	5-Fluorouracil (antimetabolite)	TGF-β signaling pathway	Drives MDR and EMT in 5-FU resistance	Sun et al. (2017)
TGF-β	Oncogene	SW480, HCT116	*In vitro* and *in vivo*	Oxaliplatin (alkylating agent)	TGF-β/PI3K/AKT	Induces anti-apoptotic signaling, oxaliplatin resistance	Tian et al. (2025)
TGF-β	Oncogene	COLO205, SW620	*In vitro* and *in vivo*	CAR-T cell therapy	TLR4/MYD88/TGF-β	Enhances stemness, induces CAR-T resistance	Tao et al. (2025)
IL-6, TGF-β2	Oncogene	HT-29, Colo205	*In vitro*	Doxorubicin (anthracycline antibiotic)	IL-6/TGF-β2	Secreted markers of doxorubicin resistance	Sritharan and Sivalingam (2024)
TGFα	Oncogene	LIM1215, OXCO-2, DiFi	*In vitro*	Cetuximab, Panitumumab (EGFR-targeted mAbs)	EGFR/ERK	Paracrine EGFR activation bypasses blockade	Hobor et al. (2014)

Cytokines such as IL-8, TGF-β1, and TNF-α significantly contribute to chemotherapy resistance by altering tumor cell dynamics and the TME. In doxorubicin-resistant CRC cell lines, IL-8 expression is markedly elevated compared to parental cells, unlike FGF-2, EGF, TGF-β, IL-6, or IL-10. IL-8 drives Dox resistance by upregulating multidrug resistance 1 (MDR1) through the IKK-β/p65 signaling pathway, enhancing drug efflux. Targeted inhibition of IL-8 using siRNAs or reparixin restores Dox sensitivity by reducing MDR1 expression without affecting ABCC1. NF-κB inhibitors, such as BAY 11–7082, and IKK-β inhibitors, like ACHP, further suppress IL-8-induced MDR1 upregulation, confirming IL-8’s pivotal role in chemotherapy resistance (Du et al., 2018). Similarly, TGF-β1 promotes oxaliplatin resistance in CRC cell lines by inducing EMT. Exposure to TGF-β1 diminishes DNA damage and apoptosis triggered by oxaliplatin, thereby reducing its efficacy. This EMT-mediated mechanism underscores TGF-β1’s role in shielding tumor cells from chemotherapy-induced death (Mao et al., 2017). Additionally, TNF-α, alongside IL-1 and IL-6, contributes to oxaliplatin resistance through the circular RNA hsa_circ_0079662. Overexpressed in oxaliplatin-resistant cells, this circRNA acts as a competing endogenous RNA (ceRNA), binding hsa-mir-324-5p to upregulate HOXA9 and TRIP6 via the TNF-α pathway. This cascade elevates pro-tumorigenic proteins, including Vcam-1, VEGFC, MMP3, MMP9, and MMP14, fostering a resistant TME. These cytokines collectively create a protective shield for CRC cells, highlighting the potential of cytokine-targeted therapies to enhance chemotherapy efficacy (Lai et al., 2020). In immunotherapy, particularly immune checkpoint blockade, cytokines like IL-6 and IFN-γ, and chemokines like CXCL8, profoundly influence resistance by modulating immune cell activity within the TME. Among 105 CRC patients, high IL-6 expression in 60 tumor tissues correlated with shorter survival (median 25.5 months) compared to low IL-6 expression (median 46 months). In CRC mouse models using CT26 and MC38 cells, IL-6 overexpression accelerates tumor growth, reduces CD8^+^ and CD4^+^ T cell infiltration, and increases myeloid-derived suppressor cells and regulatory T cells. This immunosuppressive milieu upregulates PD-L1, contributing to IL-6-driven resistance to anti-PD-L1 therapy. Notably, IL-6 blockade enhances anti-PD-L1 efficacy, extending survival in tumor-bearing mice, positioning IL-6 as a key mediator of immunotherapy resistance (Li et al., 2018).Likewise, downregulation of IFN-γ receptor (IFNGR1) in MC38 cells induces resistance to anti-PD-1 therapy in a C57BL/6 xenograft model. IFNGR1 knockdown reduces TILs, impairing antitumor immunity and allowing resistant and sensitive tumor cells to grow in distinct spatial regions, unaffected by anti-PD-1 treatment. IFN-γ signaling is thus essential for immunotherapy sensitivity, and its disruption promotes immune evasion (Lv et al., 2021). Furthermore, CXCL8 (IL-8) and its receptors CXCR1/2, upregulated in chemoresistant HCT116 sublines, are stimulated by IL-1α via IL-1 receptor signaling. This enhances cell proliferation and immune suppression, reinforcing CXCL8’s role in resistance to immune checkpoint inhibitors, particularly in microsatellite-stable CRC, where efficacy is often limited (Dabkeviciene et al., 2015).

In targeted therapies, such as anti-angiogenic treatments, chemokines like CXCL12 and cytokines like TNF-α and IFN-γ critically regulate resistance mechanisms. Anti-VEGFR2 therapies, such as ramucirumab, upregulate CXCL12 and its receptor CXCR4 in orthotopic CRC models, recruiting immunosuppressive Ly6Clow monocytes that undermine treatment efficacy. CXCR4 blockade enhances anti-VEGFR2 therapy by preventing this recruitment, with selective depletion of Ly6Clow monocytes or neutrophils yielding similar improvements in SL4 tumors, though neutrophil depletion is less effective in CT26 tumors. CXCL12/CXCR4 signaling emerges as a central driver of resistance to anti-VEGF therapies, offering a promising strategy to improve therapeutic outcomes (Jung et al., 2017). Additionally, TNF-α and IFN-γ synergize to overcome resistance to TRAIL therapy, a targeted approach leveraging immune effector molecules. In metastatic colon carcinoma cells, these cytokines sensitize cells to TRAIL-induced apoptosis by repressing Bcl-xL and Survivin expression and enhancing caspase-8 activation. *In vivo*, TRAIL therapy combined with TNF-α/IFN-γ-producing cytotoxic T lymphocytes effectively suppresses colon carcinoma metastasis, highlighting the synergistic role of these cytokines in enhancing TRAIL-based targeted therapies. This cooperative mechanism underscores their potential in overcoming resistance in metastatic CRC (Liu et al., 2011).

Chemokines and cytokines are integral to therapeutic resistance in CRC, shaping responses to chemotherapy, immunotherapy, and targeted therapies. IL-8, TGF-β1, and TNF-α drive chemotherapy resistance by promoting drug efflux, EMT, and pro-tumorigenic signaling. In immunotherapy, IL-6, IFN-γ, and CXCL8 foster immunosuppression and limit TIL infiltration, reducing checkpoint inhibitor efficacy. For targeted therapies, CXCL12/CXCR4 and TNF-α/IFN-γ modulate resistance to anti-VEGF and TRAIL therapies by altering immune cell recruitment and tumor cell survival. These interconnected mechanisms highlight the therapeutic potential of targeting specific chemokines and cytokines to overcome resistance, paving the way for innovative combination strategies to enhance CRC treatment outcomes.

### 3.3 Growth factors

Among the most prominent are members of the FGF family and VEGF, both of which contribute to resistance mechanisms against chemotherapy and targeted therapies (Zahra et al., 2021). Accumulating evidence highlights the involvement of various FGFs in conferring resistance to standard chemotherapeutic agents such as 5-FU and cisplatin. FGF2 has been shown to promote resistance to 5-FU in colon cancer cells via activation of the PI3K/Akt/mTOR signaling cascade. In 5-FU–resistant HCT116-R cells, elevated levels of FGF2 and its receptor FGFR1 were observed, accompanied by increased phosphorylation of PI3K, Akt, and mTOR, all of which are critical mediators of cell survival and drug resistance. Conversely, inhibition of FGFR signaling using AZD4547 significantly reduced chemoresistance, indicating that FGF2 facilitates chemoresistance by stimulating survival-promoting pathways downstream of PI3K/Akt (Jian et al., 2023). Similarly, FGF9 plays a central role in mediating resistance to cisplatin. Studies using the LoVo colorectal cancer cell line demonstrated that exogenous FGF9 reduced cisplatin-induced apoptosis, while its silencing enhanced chemosensitivity. Mechanistically, FGF9 activated the Wnt/β-catenin pathway by suppressing APC expression, thereby sustaining β-catenin accumulation and contributing to drug resistance. Notably, increased FGF9 expression was observed in cisplatin-resistant LoVo cells, and silencing this growth factor reversed their resistance phenotype (Zhang Z. et al., 2020).

Beyond conventional chemotherapy, growth factors are also critically involved in mediating resistance to targeted therapies, particularly anti-EGFR and anti-angiogenic agents. FGF9 has again been implicated in resistance to anti-EGFR therapies, such as cetuximab. Clinical analyses revealed that FGF9 gene amplification and overexpression were more frequent in non-responders, especially those with wild-type KRAS. Functional studies demonstrated that FGF9 overexpression activates FGFR signaling, leading to robust resistance to anti-EGFR agents, which could be effectively reversed using FGFR inhibitors. These findings underscore the relevance of FGF9 as a driver of anti-EGFR therapy resistance in CRC, and suggest that combined FGFR inhibition may restore therapeutic efficacy (Mizukami et al., 2017). In addition, VEGF-mediated autocrine signaling has been linked to resistance against bevacizumab, a monoclonal antibody targeting VEGF. Bevacizumab-resistant CRC cells exhibited upregulated HIF-VEGF-VEGFR signaling, increased hypoxia tolerance, and enhanced cell survival, highlighting the role of autocrine VEGF loops in maintaining resistance to anti-angiogenic therapy. Importantly, these resistant models remained sensitive to nintedanib, a multi-kinase angiogenesis inhibitor that suppresses mTORC1 signaling. Nintedanib was found to be particularly effective under hypoxic conditions, suggesting it could overcome hypoxia-induced resistance by disrupting VEGF-dependent survival mechanisms (Mésange et al., 2014; Wang et al., 2018). Collectively, these findings highlight the multifaceted roles of FGF and VEGF family members in promoting resistance to both chemotherapy and targeted therapies in colorectal cancer. By activating key survival pathways such as PI3K/Akt/mTOR, Wnt/β-catenin, and autocrine VEGF signaling, growth factors shield CRC cells from therapeutic insults. Targeting these signaling axes through FGFR or angiokinase inhibitors presents a promising strategy to restore drug sensitivity and improve clinical outcomes in resistant colorectal cancer.

Non-cellular elements of the tumor microenvironment, extracellular matrix remodeling, hypoxic niches, and dysregulated soluble factors, act in concert with cellular components to sustain resistance. Hypoxia and TGF-β drive metabolic shifts and epithelial-mesenchymal transition that favor cancer stemness and drug efflux, while stiffened ECM and cytokine networks, IL-6, VEGF, promote angiogenesis and immune exclusion. These processes reinforce cellular immunosuppression by recruiting and polarizing myeloid cells, suppressing effector lymphocytes, and limiting drug penetration. Targeting these non-cellular drivers, through hypoxia modulators, ECM-degrading agents, or broad cytokine inhibition, therefore holds promise to disrupt the supportive scaffold of the TIME and enhance the efficacy of conventional and immune-based treatments when combined with cellular reprogramming strategies.

## 4 Metabolic factors

### 4.1 Hypoxia

By promoting metabolic reprogramming and an immunosuppressive TME, hypoxia fosters resistance through molecular pathways, miRNA regulation, and immune modulation. Hypoxia markedly reduces chemotherapy sensitivity to agents like oxaliplatin and 5-FU (Fletcher et al., 2022). In CRC cell lines under cobalt chloride-induced hypoxia, enhanced glycolysis diminishes oxaliplatin efficacy via the HIF-1α/BMAL1/ALDOC axis. HIF-1α upregulates BMAL1, increasing ALDOC expression, which boosts glycolytic activity and suppresses apoptosis. Clinical CRC samples confirm a positive HIF-1α/ALDOC correlation, suggesting their role as predictive biomarkers (Ran et al., 2025). Similarly, hypoxia fuels 5-FU resistance by enhancing glycolysis and the pentose phosphate pathway, driven by HIF-1α upregulation through ROS-mediated PI3K/Akt signaling and β-catenin activation. Genetic or pharmacological HIF-1α inhibition restores 5-FU sensitivity (Dong et al., 2022; Tong et al., 2022). Additionally, a hypoxia-induced HIF-1α/miR-338-5p/IL-6 feedback loop promotes oxaliplatin resistance by activating STAT3/Bcl2 signaling. In xenograft models, overexpressing miR-338-5p or using HIF-1α inhibitor PX-478 enhances oxaliplatin sensitivity, underscoring the therapeutic potential of targeting this loop (Xu et al., 2019).

Hypoxia creates an immunosuppressive TME, limiting immunotherapy efficacy in CRC, particularly in CRLM. In high-metastatic CRC cell lines, LoVo-HM, HCT116-HM, spatial transcriptomics revealed hypoxia-driven SPP1 upregulation, which activates β-catenin/HIF-1α signaling, increasing CXCL12 secretion by CAFs. This SPP1/CAF/CXCL12 axis reduces CD8^+^ T cell infiltration and IFN-γ+/GZMB+ T cell activity, fostering immunotherapy resistance. Targeting SPP1 and CXCL12 with Plerixafor in mouse models enhances immunotherapy efficacy by boosting T cell infiltration, leading to metastatic lesion regression, highlighting hypoxia’s role in immune suppression (Liu S. et al., 2025).

Hypoxia also drives resistance to targeted therapies, such as EGFR-targeted Cetuximab in RAS wild-type metastatic CRC. Elevated circHIF1A in Cetuximab-resistant LIM1215-R cells upregulates HIF-1α by binding miR-361-5p, increasing GLUT1 and LDHA expression, which enhances glycometabolism and resistance. Inhibiting circHIF1A restores Cetuximab sensitivity in xenograft models, suggesting its use as a biomarker (Geng et al., 2024). Conversely, hypoxia-downregulated circ_0001766 inhibits CRC progression by sponging miR-1203, stabilizing PPP1R3C mRNA, and suppressing mTOR/Myc signaling. Reduced circ_0001766 levels under hypoxia promote rapamycin resistance, but its overexpression resensitizes cells, offering a strategy to overcome resistance. These findings emphasize hypoxia’s modulation of circRNA-mediated pathways in targeted therapy resistance (Zhou et al., 2025a; Xiao et al., 2020).

Hypoxia’s central role in driving resistance through metabolic reprogramming, molecular feedback loops, and immune suppression links chemotherapy, immunotherapy, and targeted therapy challenges (Table 8). The HIF-1α/BMAL1/ALDOC, HIF-1α/miR-338-5p/IL-6, SPP1/CAF/CXCL12, and circRNA-mediated pathways converge on hypoxia’s ability to enhance glycolysis and foster an immunosuppressive TME. Targeting HIF-1α or downstream mediators like SPP1, CXCL12, or circRNAs with inhibitors such as PX-478 or Plerixafor offers synergistic potential to restore treatment sensitivity, paving the way for personalized CRC therapies.

**TABLE 8:** Hypoxia and HIFs in colorectal cancer: Mechanisms of chemoresistance.

Hypoxia or its factors	Role of hypoxia	Regulator of hypoxia	Cell lines	Type of study	Therapeutic type	Axis	Highlightes	References
HIF-1α	Oncogene	cobalt chloride (CoCl2)	LOVO	*In vitro*	5-Fluorouracil (antimetabolite)	HIF-1α/MDR1, MRP	CoCl2-induced chemoresistance and reduced apoptosis	Yang et al. (2016)
Hypoxia	Oncogene	​	HCT116, SW480	*In vitro*	5-Fluorouracil (antimetabolite)	miR-675-5p/Caspase-3	miR-675-5p inhibits caspase-3, reduces apoptosis	Zichittella et al. (2022)
HIF-1α	Oncogene	​	HT29, SW480	*In vitro*	5-Fluorouracil (antimetabolite), Oxaliplatin (alkylating agent)	​	Induces G0/G1 arrest; SN-38 overcomes resistance	Murono et al. (2012)
Hypoxia	Oncogene	​	LoVo, HT-29, HCT116	*In vitro*, *In vivo*	Oxaliplatin (alkylating agent)	HOTAIR/miR-1277-5p/ZEB1	HOTAIR axis regulates EMT in resistance	Weng et al. (2022)
Hypoxia	Oncogene	​	LoVo, HT29, HCT116	*In vitro*, *In vivo*	Oxaliplatin (alkylating agent)	H19/miR-675-3p/EMT	H19 downregulation counteracts EMT resistance	Weng et al. (2024)
HIF-1α	Oncogene	miR-495-3p	HCT116, SW480	*In vitro*	5-Fluorouracil (antimetabolite)	NORAD/miR-495-3p/HIF-1α	NORAD knockdown reduces VM and EMT	Zhang et al. (2022a)
HIF-1α	Oncogene	ROS/PI3K/Akt, Wnt/β-catenin	HCT8, HCT15, LoVo	*In vitro*, *In vivo*	5-Fluorouracil (antimetabolite)	HIF-1α/GLUT1, HK2, PKM2, LDHA, MCT4	Activates metabolic reprogramming for resistance	Dong et al. (2022)

### 4.2 Lactate accumulation (warburg effect)

A defining metabolic hallmark of CRC is the Warburg effect, wherein cancer cells preferentially convert glucose into lactate even under normoxic conditions. This altered metabolism not only fuels tumor growth but also orchestrates a complex network of resistance mechanisms that limit the efficacy of both chemotherapy and targeted therapies (Yu et al., 2023).

Oxaliplatin, a cornerstone chemotherapeutic agent in CRC, is notably impacted by lactate-driven resistance mechanisms. CAFs, reprogrammed to favor glycolysis, secrete high levels of lactate into the tumor microenvironment. This extracellular lactate enters CRC cells and facilitates histone lactylation, particularly promoting the transcription and lactylation of ANTXR1 at lysine 453. The resultant upregulation of ANTXR1 and its lactylated form enhances activation of the RhoC/ROCK1/SMAD5 signaling cascade, reinforcing stemness characteristics and oxaliplatin resistance. Notably, disrupting the lactate shuttle between CAFs and cancer cells, either genetically or pharmacologically, has shown promising results in restoring oxaliplatin sensitivity in preclinical models (He et al., 2025). In parallel, 5-FU resistance in CRC is closely tied to enhanced glycolytic flux. Resistant cells exhibit elevated ATP production, glucose consumption, and lactate secretion. At the molecular level, this phenotype is driven by upregulation of METTL3, an m6A RNA methyltransferase. METTL3 stabilizes HIF-1α mRNA and enhances the translation of LDHA, the enzyme responsible for converting pyruvate to lactate. The METTL3/LDHA axis thereby fuels glycolysis, sustaining the metabolic state necessary for 5-FU resistance. Targeted inhibition of METTL3 or LDHA re-sensitizes CRC cells to 5-FU, presenting a promising therapeutic strategy (Zhang K. et al., 2022). In the metastatic setting, particularly in liver metastases, the tumor microenvironment further amplifies lactate-mediated resistance. HSCs, upon stimulation by CRC-derived exosomes, release IL-6, which activates the IL-6/STAT3 pathway in hypoxic CRC cells. This axis upregulates MCT1 and LDHB, key regulators of lactate uptake and utilization, thereby enhancing lactate metabolism and conferring resistance to SN38, the active metabolite of irinotecan (Li et al., 2020).

Beyond chemotherapy, lactate metabolism is intricately linked to resistance against targeted therapies. In cetuximab-resistant CRC models harboring KRAS mutations, a pronounced increase in glycolytic activity and lactate recycling has been observed. These cells overexpress MCT1, which facilitates lactate import to support continuous metabolic demands. Pharmacological inhibition of MCT1 with agents like AR-C155858 significantly impairs lactate utilization, suppressing tumor growth both *in vitro* and *in vivo*. Thus, MCT1-dependent lactate utilization emerges as a targetable vulnerability in overcoming anti-EGFR therapy resistance (Richiardone et al., 2024). Resistance to bevacizumab, a widely used antiangiogenic agent, is also influenced by hypoxia-induced lactate accumulation. Under hypoxic conditions, increased glycolysis elevates intracellular lactate, which promotes histone lactylation. Specifically, lactylation at H3K18 upregulates RUBCNL/Pacer, facilitating autophagosome maturation through interaction with BECN1 and activation of the class III PI3K complex. This adaptation enables CRC cells to thrive under bevacizumab-induced hypoxic stress. Co-inhibition of lactylation and autophagy synergizes with bevacizumab to overcome resistance in patient-derived models, underscoring the significance of lactate-mediated epigenetic reprogramming in modulating therapeutic outcomes (Li W. et al., 2024).

Collectively, these findings highlight the central role of lactate accumulation and metabolism in driving resistance to both chemotherapeutic and targeted agents in CRC. Lactate not only serves as a metabolic byproduct but also functions as a signaling molecule that reprograms cancer and stromal cells via epigenetic and paracrine mechanisms. Targeting lactate production, transport, and utilization, particularly through inhibition of LDHA, METTL3, MCT1, or histone lactylation, represents a promising avenue to reverse resistance and improve therapeutic efficacy in colorectal cancer.

### 4.3 Nutrient competition

Among the various metabolic resources, amino acids and glucose have emerged as critical modulators, shaping tumor progression, immune evasion, and resistance to chemotherapy, immunotherapy, and targeted treatments. The aggressive metabolic reprogramming of CRC cells not only enhances their survival and proliferation but also deprives immune cells of essential nutrients, thereby weakening antitumor responses and compromising therapeutic efficacy (Lewandowska et al., 2022).

One of the major contributors to chemotherapy resistance in CRC is the rewiring of glucose and amino acid metabolism, which supports tumor cell survival and interferes with drug activity. A key player in this process is the long noncoding RNA LINC01764, which enhances glycolysis and glutamine metabolism, ultimately promoting tumor growth, metastasis, and resistance 5-FU. Mechanistically, LINC01764 binds to hnRNPK, facilitating the translation of c-MYC via an internal ribosome entry site (IRES)-dependent mechanism. This activation of the c-MYC/glucose/glutamine axis suppresses UPP1, an enzyme essential for 5-FU activation, thereby weakening its chemotherapeutic effect (Duan R. et al., 2024). Glucose deprivation induces ATF4 expression, enhancing MDR1 and protecting cells from oxaliplatin and 5-FU-induced apoptosis (Hu et al., 2016). Inhibiting ATF4 reverses this resistance. Additionally, AKT1/3 activation in resistant CRC cells suppresses miR-125b-5p, leading to increased GLUT5 expression, metabolic reprogramming, and resistance. Restoration of miR-125b-5p or GLUT5 inhibition re-sensitizes these cells (Park et al., 2020). Clinically, hyperglycemia correlates with poor prognosis in stage III CRC patients on FOLFOX4 therapy. High glucose levels enhance oxaliplatin resistance via SMAD3 and MYC phosphorylation, while metformin restores drug sensitivity. Together, these findings underscore the role of glucose and amino acid metabolism in mediating CRC chemoresistance and highlight metabolic targeting as a promising strategy (Yang et al., 2019). In addition to glucose and glutamine, methionine metabolism also plays a central role in mediating chemoresistance. Dietary methionine restriction has been shown to significantly enhance the sensitivity of colorectal cancer stem cells (CRC-SCs) to 5-FU. In chemoresistant CD133^+^ CRC cells, the combination of methionine restriction and 5-FU treatment not only reduced tumor cell viability and tumor volume but also downregulated ABCG2, a key drug-efflux transporter. This sensitization is driven by the upregulation of miR-320d, which inhibits c-MYC expression and, in turn, diminishes the chemoresistant phenotype. These findings highlight how targeting amino acid metabolism can reverse resistance in CRC-SCs and improve chemotherapeutic outcomes (Liu C. et al., 2022).

The influence of nutrient metabolism extends beyond chemotherapy and significantly impacts response to immunotherapy. Methionine, in particular, modulates the immune landscape within the tumor microenvironment. Methionine restriction downregulates the expression of PCSK9, a regulator of cholesterol metabolism and immune signaling, thereby enhancing CD8^+^ T cell infiltration and significantly improving the efficacy of PD-1 immune checkpoint blockade in MSS CRC models, tumors that are typically unresponsive to such therapies. Notably, the combination of methionine restriction with 5-FU and PD-1 inhibition resulted in synergistic antitumor effects, offering a compelling strategy for MSS CRC treatment (Wang Q-L. et al., 2025). On a mechanistic level, methionine restriction activates the cGAS-STING–interferon signaling pathway, which leads to increased expression of MHC-I and PD-L1 on CRC cells. This transformation enhances tumor immunogenicity and augments the response to immune checkpoint inhibitors, including anti-CTLA-4 and anti-PD-1 therapies. The improved immune response is accompanied by a shift in CD8^+^ T cell localization, from the tumor periphery to the tumor core, indicating deeper immune infiltration and greater antitumor activity. These findings underscore the immunomodulatory power of amino acid metabolism, particularly methionine, in overcoming resistance to immunotherapy (Morehead et al., 2023).

Metabolic alterations can also affect the efficacy of targeted therapies. For example, resistance to PARP inhibitors like olaparib in BRCA-functional CRC cells may be influenced by nutrient-sensitive transcriptional regulation. Although not directly tied to nutrient competition, these pathways often converge with metabolic signals. For instance, the expression of BRCA2 is regulated by histone acetylation, a process influenced by intracellular metabolic status. In this context, nutrient-driven pathways, such as those involving c-MYC or S-adenosylmethionine, may indirectly modulate responses to DNA repair-targeting agents (Chen and Allgayer, 2023). Furthermore, the upregulation of GLUT3, a glucose transporter encoded by the SLC2A3 gene, has been associated with acquired resistance to both butyrate and histone deacetylase inhibitors like trichostatin A (TSA). In butyrate-resistant CRC cells, long-term exposure led to over a 20-fold increase in SLC2A3 expression and a two-fold rise in GLUT3 protein levels. Silencing SLC2A3 restored sensitivity to TSA, confirming that enhanced glucose uptake via GLUT3 contributes to metabolic adaptation and targeted therapy resistance (Khonthun and Surangkul, 2023).

In summary, amino acids, specifically methionine and glutamine, and glucose are not merely nutritional resources but central players in shaping drug resistance in CRC. By fueling oncogenic signaling, impairing immune function, and altering the tumor microenvironment, these nutrients critically influence the success of chemotherapy, immunotherapy, and targeted therapies. Therapeutic strategies aimed at disrupting nutrient uptake and metabolism, such as dietary restriction, transporter inhibition, or modulation of metabolic gene expression, hold great promise for reversing resistance and improving outcomes in colorectal cancer patients.

## 5 Therapeutic approaches of targeting TME in CRC drug resistance

### 5.1 Natural compounds

Key TME factors, including M2-polarized macrophages, hypoxia, EMT, and immune suppression, drive chemoresistance, limiting treatment efficacy. Natural compounds have emerged as powerful tools to counteract these mechanisms, offering innovative strategies to enhance chemosensitivity in CRC by targeting the TME. Derived often from traditional Chinese medicine, these compounds synergize with conventional therapies, paving the way for improved clinical outcomes (Elimam et al., 2025; Kim et al., 2024).

Bufalin, the primary active component of Cinobufacini, demonstrates significant potential in reversing CRC chemoresistance by modulating M2 macrophage polarization within the TME. Research shows that Bufalin diminishes M2 polarization triggered by chemoresistant CRC cells both *in vitro* and *in vivo*, primarily by targeting the SRC-3 protein to reduce macrophage MIF release. This disruption curbs the pro-tumorigenic M2 macrophage phenotype, amplifying the anti-tumor effects of oxaliplatin in preclinical and clinical settings. Moreover, Bufalin counters hypoxia-driven resistance by lowering SRC-3 and HIF-1α levels, which are elevated in hypoxic CRC cells and promote M2 polarization. By alleviating hypoxia, Bufalin restores chemotherapy sensitivity, establishing itself as a promising adjuvant therapy for CRC (Chen et al., 2021c; Wang H. et al., 2025). Building on this, andrographolide sulfonate enhances 5-FU efficacy by mitigating immune suppression within the TME. In preclinical models, combining andrographolide sulfonate with 5-FU markedly suppressed tumor growth in CT26 colon cancer transplants, promoting apoptosis and inhibiting proliferation. This combination boosted CD4^+^ and CD8^+^ T cell infiltration and upregulated IFN-γ and Granzyme B expression, reflecting enhanced antitumor immunity. Andrographolide sulfonate’s ability to reverse 5-FU-induced NLRP3 inflammasome activation in MDSCs further sensitizes CRC cells to chemotherapy, highlighting its role in overcoming immune-mediated resistance (Xu L. et al., 2021).

Similarly, Curcumin, a well-researched natural compound, effectively reverses oxaliplatin resistance by inhibiting the NF-κB signaling pathway, a critical driver of chemoresistance. In OXA-resistant CRC cell lines, Curcumin downregulates NF-κB-regulated CXC-chemokines, CXCL8, CXCL1, and CXCL2, disrupting pro-tumorigenic signaling. Additionally, Curcumin suppresses TGF-β/Smad-mediated EMT, a key mechanism of oxaliplatin resistance, by reducing p-p65 and Bcl-2 expression while increasing active-caspase3 levels. *In vivo* studies confirm that combining Curcumin with oxaliplatin enhances treatment efficacy, with elevated CXCL1 expression in patient-derived liver metastases serving as a predictive marker for responsiveness. Curcumin’s dual modulation of NF-κB and TGF-β pathways underscores its versatility in tackling TME-driven resistance (Ruiz de Porras et al., 2016; Yin et al., 2019). Pien Tze Huang (PZH), a traditional Chinese medicine formula, addresses MDR and EMT in 5-FU-resistant CRC cells. PZH dose-dependently inhibits the viability of HCT-8/5-FU cells, reduces ABCG2 expression, and enhances intracellular Rhodamine-123 accumulation, countering MDR. By suppressing TGF-β signaling, PZH reverses EMT-related morphological changes, curbing migration and invasion in resistant cells. PZH’s comprehensive action on MDR and EMT highlights its potential to restore chemosensitivity within the TME (Shen et al., 2014). Coptidis Rhizoma extract (CRE) further exemplifies the anti-metastatic and anti-EMT capabilities of natural compounds in drug-resistant CRC. In 5-FU-resistant HCT116/R cells, CRE upregulates E-cadherin and downregulates vimentin, Snail, and ZEB2, effectively inhibiting EMT-driven invasion and migration. This effect is mediated through suppression of TGF-β signaling, as evidenced by reduced phosphorylation of Akt and p38. CRE’s targeted inhibition of TGF-β-mediated EMT positions it as a promising candidate for mitigating resistance and metastasis in CRC (Kang et al., 2022; Duan W-W. et al., 2024).

Pentagalloyl glucose (PGG), derived from Bouea macrophylla seeds, targets CSCs and EMT in 5-FU-resistant CRC by inhibiting the JAK1/JAK3-STAT3 signaling pathway. PGG reduces CSC markers, CD133, CD44, EMT regulators, N-cadherin, vimentin, and anti-apoptotic proteins Bcl-2, promoting apoptosis and sensitizing resistant cells to 5-FU. Across 2D, 3D, and xenograft models, PGG exhibits robust anti-tumor effects, underscoring its role in overcoming TME-driven resistance by disrupting CSC and EMT dynamics (Wen et al., 2025). Rediocide-A (Red-A), a natural immune checkpoint inhibitor, enhances NK cell-mediated tumoricidal activity within the TME. By downregulating CD155 expression in cancer cells, Red-A overcomes immune resistance, increasing NK cell lysis by up to 3.58-fold and elevating IFN-γ and Granzyme B production. Red-A’s modulation of TIGIT/CD155 signaling highlights its potential as an immunotherapy adjuvant, complementing chemotherapeutic approaches in CRC (Ng et al., 2021).

**FIGURE 2:**
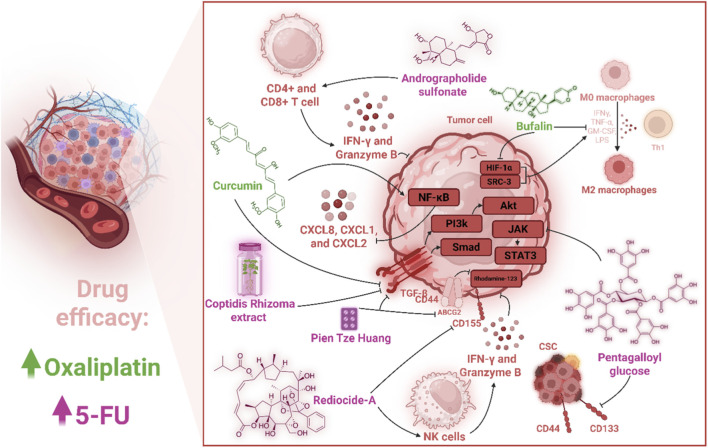
Natural Compounds Targeting the TME to Overcome Chemoresistance in Colorectal Cancer. Natural compounds derived largely from traditional Chinese medicine enhance chemosensitivity in CRC by modulating the TME. These agents counteract key resistance drivers such as M2 macrophage polarization, hypoxia, EMT, CSCs, and immune suppression. Bufalin targets SRC-3 and HIF-1α to inhibit M2 polarization and hypoxia-driven resistance, enhancing oxaliplatin efficacy. Andrographolide sulfonate reverses 5-FU resistance by boosting CD4+/CD8+ T cell activity and mitigating immune suppression. Curcumin inhibits NF-κB and TGF-β/Smad signaling to block EMT and chemoresistance, while Pien Tze Huang suppresses multidrug resistance (MDR) and EMT by downregulating ABCG2. Coptidis Rhizoma extract disrupts TGF-β-mediated EMT, reducing invasion and metastasis. Pentagalloyl glucose impairs CSC and EMT pathways via JAK/STAT3 inhibition, restoring 5-FU sensitivity. Rediocide-A strengthens NK cell-mediated cytotoxicity by targeting TIGIT/CD155 signaling. Together, these compounds act on multiple molecular targets to overcome chemoresistance, enhance oxaliplatin and 5-FU efficacy, and promote improved therapeutic outcomes in CRC.

Altogether, these findings underscore that natural compounds provide a multifaceted approach to overcoming CRC chemoresistance by targeting critical TME components, including M2 macrophages, hypoxia, EMT, CSCs, and immune suppression. By modulating key pathways such as TGF-β, NF-κB, and JAK/STAT3, these compounds enhance the efficacy of 5-FU and oxaliplatin, restore immune surveillance, and inhibit metastatic potential (Figure 2). Their synergistic effects and ability to address complex resistance mechanisms position natural compounds as transformative agents for future clinical strategies in CRC treatment.

### 5.2 Probiotics and microbiota

**FIGURE 3:**
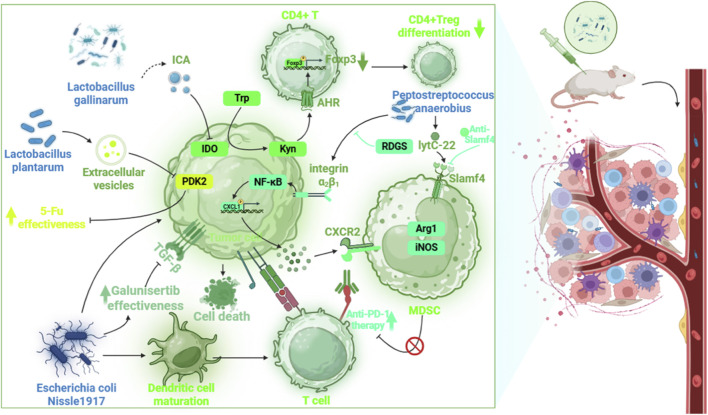
Modulation of CRC drug resistance through gut microbiota interventions. The gut microbiota influences the TME and therapy responsiveness in CRC. Probiotic-derived extracellular vesicles from *Lactobacillus plantarum* (LpEVs) counteract chemoresistance by suppressing PDK2 expression, restoring 5-FU sensitivity. *Lactobacillus gallinarum* enhances anti-PD-1 therapy by reducing regulatory T cell (Treg) differentiation through inhibition of the IDO1/kynurenine/AHR pathway. Conversely, *Peptostreptococcus anaerobius* drives immune evasion by activating integrin α2β1–NF-κB signaling in tumor cells, promoting CXCR2+ MDSC recruitment and reinforcing their immunosuppressive function via Slamf4–lytC_22 interactions. Engineered *Escherichia coli* Nissle 1917, encapsulated in chitosan-sodium alginate microgels, boosts Galunisertib’s efficacy by inducing immunogenic cell death, enhancing T cell infiltration, and promoting apoptosis. Together, these strategies underscore the therapeutic potential of microbiota modulation to improve chemotherapy, targeted therapy, and immunotherapy outcomes in CRC.

The gut microbiota plays a pivotal role in shaping the TME and represents a promising target for overcoming drug resistance in CRC. A growing body of evidence suggests that probiotics and their bioactive metabolites can modulate tumor metabolism and immunity, thereby enhancing the efficacy of chemotherapy, targeted therapies, and immunotherapies (Figure 3) (Li Q. et al., 2024).

Chemoresistance, particularly to 5-FU, remains a major barrier in CRC treatment and is closely associated with metabolic reprogramming marked by enhanced glycolysis. Recent findings have highlighted the therapeutic potential of extracellular vesicles derived from *Lactobacillus plantarum* (LpEVs) in reversing this metabolic shift. In 5-FU-resistant CRC cells, LpEVs downregulate pyruvate dehydrogenase kinase 2 (PDK2), a key glycolytic enzyme upregulated in resistant cells, via the p53-p21 signaling axis. This metabolic reprogramming leads to reduced cell proliferation and increased apoptosis, thereby resensitizing resistant CRC cells to 5-FU. These findings underscore the potential of probiotic-derived vesicles in targeting cancer cell metabolism as a novel strategy to overcome chemotherapy resistance (An and Ha, 2022).

Beyond chemotherapy, manipulating the gut microbiota has emerged as an effective approach to boost the efficacy of targeted therapies and immune checkpoint inhibitors. Notably, *L. gallinarum* has demonstrated a potent immunomodulatory role in enhancing anti-PD1 therapy in CRC. In murine models representing both MSI-H and MSI-low CRC, *L. gallinarum* significantly improved anti-PD1 responses by reducing the infiltration of immunosuppressive Tregs and strengthening the effector functions of CD8^+^ T cells within tumors. Mechanistically, its metabolite indole-3-carboxylic acid (ICA) was found to inhibit the IDO1/kynurenine (Kyn)/aryl hydrocarbon receptor (AHR) axis, a key pathway involved in Treg differentiation. By blocking this immunosuppressive signaling, ICA not only mimicked the beneficial effects of *L. gallinarum* but also significantly amplified anti-PD1 efficacy in vivo—effects that were reversed with Kyn supplementation. This highlights the potential of *L. gallinarum* and its metabolite ICA as adjuvants to potentiate immunotherapy in CRC (Fong et al., 2023). In contrast, certain pathogenic bacteria within the microbiota can impair immunotherapeutic responses and exacerbate drug resistance. *Peptostreptococcus anaerobius*, an oral commensal associated with CRC progression, has been shown to abolish the efficacy of anti-PD1 therapy by inducing the accumulation and activation of immunosuppressive MDSCs in the TME. This bacterium activates integrin α2β1–NF-κB signaling in tumor cells, leading to the secretion of CXCL1 and recruitment of CXCR2+ MDSCs. Additionally, *P. anaerobius* secretes the protein lytC_22, which directly binds to the Slamf4 receptor on MDSCs, further enhancing their suppressive functions through upregulation of ARG1 and iNOS. Therapeutically targeting integrin α2β1 or the Slamf4 receptor represents a promising strategy to reverse microbiota-induced immunotherapy resistance (Liu Y. et al., 2024).

Building on this concept, engineered probiotics offer a novel avenue for reshaping the TME and enhancing targeted therapy responses. A notable example is *Escherichia coli* Nissle 1917 (EcN), a therapeutic probiotic known to regulate gut microbiota composition and host immunity. Encapsulation of EcN in a chitosan-sodium alginate (CS-SA) microgel significantly enhanced its gastrointestinal stability and bioavailability. When combined with Galunisertib, a TGF-β inhibitor, EcN@(CS-SA)_2_ microgel promoted robust antitumor responses by inducing immunogenic cell death, enhancing CD8^+^ T cell infiltration, and increasing overall tumor apoptosis. This synergistic approach highlights the ability of engineered probiotics to reinforce immune-mediated tumor control and improve the efficacy of targeted therapies (Niu et al., 2024).

### 5.3 Nanoparticles

Recent advancements in nanotechnology have offered promising avenues to overcome this therapeutic challenge by enhancing drug delivery, modulating immune responses, and reprogramming the hostile TME. Nanoparticles have emerged as powerful tools in this context, demonstrating significant potential in circumventing resistance mechanisms and restoring therapeutic efficacy (Narayana et al., 2024; Jiang et al., 2025; Li X-P. et al., 2023).

Chemoresistance, particularly to agents like 5-FU, poses a significant obstacle in CRC treatment. Acquired drug resistance often results from the overexpression of enzymes such as dihydropyrimidine dehydrogenase, which metabolizes 5-FU into its inactive form, thereby diminishing its cytotoxic effects. To address this, researchers have engineered EGF-grafted hollow mesoporous silica nanoparticles (HMSNs) for the targeted delivery of 5-FU. These EGF-HMSNs exploit the overexpression of EGFR on resistant CRC cells, particularly the SW480/ADR line, facilitating selective cellular uptake via receptor-mediated endocytosis. Once internalized, these nanocarriers not only evade lysosomal degradation but also achieve elevated intracellular drug accumulation. As a result, EGF-HMSNs loaded with 5-FU (EGF-HMSNs-5-FU) demonstrated significantly higher cytotoxicity compared to both free 5-FU and non-targeted HMSNs-5-FU. Mechanistically, their effect was linked to S-phase cell cycle arrest, leading to enhanced cell death. This nanoparticle-mediated strategy directly targets the chemoresistance-driving elements of the TME, offering a refined approach to re-sensitize CRC cells to conventional chemotherapy (Chen et al., 2015; Li C. et al., 2024).

While immunotherapy has revolutionized oncology, its efficacy in CRC, particularly in MSS tumors, remains limited due to poor CTL infiltration and immune tolerance. To combat this, novel nanoparticle-based immunomodulatory strategies have been developed to reprogram the immune landscape of the TME. One such approach utilizes Selenium Nanoparticles-Loaded Extracellular Vesicles combined with Interleukin-32 and engineered probiotic Escherichia coli Nissle 1917 (SeNVs@NE-IL32-EcN). This multifunctional construct not only enhances CD8^+^ T cell-mediated immune responses but also mitigates immunotherapy resistance. Integrated transcriptomic analyses identified IL32 as a key enhancer of T cell cytotoxicity through granzyme B and IFN-γ pathways. In humanized xenograft models, SeNVs@NE-IL32-EcN markedly improved T cell infiltration and tumor suppression, illustrating the potency of nanoparticles in reshaping immune dynamics within the TME (Li S. et al., 2025). Another innovative design targeting MSS-CRC involves zeolitic imidazolate frameworks (ZIFs) cloaked with apoptotic body membranes, facilitating the co-delivery of ginsenoside Rg1 and atractylenolide-I (Ab@Rg1/Att-ZIF). These nanoparticles “hitchhike” via Ly-6C^+^ monocytes to reach the tumor core, where they disassemble and unleash their cargo. Rg1 fosters dendritic cell maturation and antigen presentation, while Att boosts MHC-I expression by activating the 26S proteasome. This dual action enhances CTL recognition and infiltration, dramatically improving the response to PD-1 blockade from ≈5% to ≈69%, a striking demonstration of how nanoparticles can synergistically remodel the TME to overcome immunotherapy resistance (Ye et al., 2024).

**FIGURE 4:**
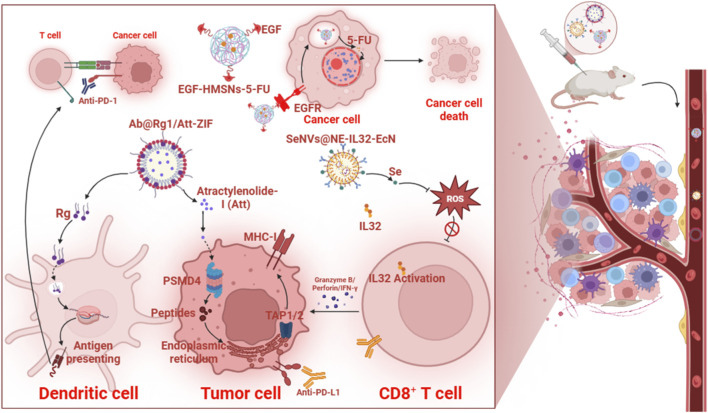
Nanoparticle-mediated strategies for overcoming therapeutic resistance in CRC. EGF-functionalized hollow mesoporous silica nanoparticles (EGF-HMSNs-5-FU) enhance intracellular delivery of 5-FU, bypassing chemoresistance mechanisms and inducing cancer cell death. Selenium-loaded extracellular vesicles combined with IL32 and engineered *E. coli* Nissle 1917 (SeNVs@NE-IL32-EcN) boost CD8^+^ T cell cytotoxicity via granzyme B, perforin, and IFN-γ, while reducing ROS-mediated immune suppression. Apoptotic body membrane-coated ZIF nanoparticles co-delivering ginsenoside Rg1 and atractylenolide-I (Ab@Rg1/Att-ZIF) promote dendritic cell maturation, increase antigen presentation, and upregulate MHC-I expression through PSMD4 and TAP1 pathways, thereby sensitizing tumors to PD-1/PD-L1 blockade. Collectively, these nanoplatforms remodel the tumor microenvironment and restore anti-tumor immune responses in resistant CRC.

Collectively, as seen in Figure 4, these studies underscore the transformative role of nanoparticles in overcoming therapeutic resistance in CRC by precisely targeting and modulating the tumor immune microenvironment. Whether by enhancing intracellular drug accumulation in chemoresistant cells or by restoring immune surveillance in immunotherapy-refractory tumors, nanoparticle-based platforms represent a versatile and promising strategy to enhance CRC treatment outcomes.

### 5.4 Pharmacological compounds

Pharmacological compounds have emerged as critical tools in modulating the TME to overcome resistance mechanisms, targeting cellular components such as CAFs, TAMs, and immunometabolic pathways (Liu Z. et al., 2024). By addressing the complex interplay between tumor cells and their stromal counterparts, these compounds offer novel strategies to enhance treatment outcomes in CRC. This section explores the role of pharmacological compounds in counteracting resistance through anti-EGFR therapy, immunotherapy combinations, and targeted inhibition of tryptophan metabolism, highlighting their immunomodulatory and resistance-reversing effects (Shakhpazyan et al., 2023).

CAFs, prevalent in the CRC stroma, express EGFR and contribute to chemotherapeutic resistance. Cetuximab, an EGFR-targeting monoclonal antibody used with chemotherapy for metastatic CRC, inadvertently induces CAFs to secrete EGF, sustaining MAPK signaling in cancer cells and conferring resistance. This CAF-specific response, absent in cancer cells or normal fibroblasts, highlights cetuximab’s role as a pharmacological compound in modulating TME-driven chemoresistance, emphasizing the need to address stromal interactions for effective therapy (Garvey et al., 2020). Immunotherapy, particularly anti-PD-1 therapies, faces challenges in MSS CRC due to immunosuppressive TME elements. FTD/TPI, an oral antimetabolite combining trifluridine and tipiracil, synergizes with oxaliplatin to induce ICD in MSS CRC cell lines, CT26, SW620, and *in vivo* models. This combination depletes type-2 TAMs, boosting cytotoxic CD8^+^ T-cell infiltration while upregulating PD-L1 and PD-1, which can lead to T-cell exhaustion. However, combining FTD/TPI and oxaliplatin with anti-PD-1 enhances antitumor efficacy, demonstrating their immunomodulatory potential as pharmacological compounds to overcome immunotherapy resistance by reshaping the TME (Limagne et al., 2019). Cetuximab resistance in CRC is often linked to TME-mediated mechanisms, including activation of the IDO1-mediated tryptophan metabolism pathway, which promotes immune evasion. Epacadostat, a selective IDO1 inhibitor, counteracts this resistance by reducing kynurenine levels, inhibiting cetuximab-resistant CRC cell proliferation, and promoting apoptosis. Combined with cetuximab, it enhances CD8^+^ T-cell infiltration and M1 macrophage polarization, transforming “cold” tumors into “hot” ones. This synergistic effect underscores epacadostat’s role as a pharmacological compound in overcoming targeted therapy resistance by targeting immunometabolic pathways in the TME (Zhou et al., 2025b).

By integrating pharmacological compounds like cetuximab, FTD/TPI, oxaliplatin, and epacadostat, therapeutic strategies can address TME-mediated resistance across chemotherapy, immunotherapy, and targeted therapy. These compounds not only target tumor cells but also modulate stromal and immune components, such as CAFs, TAM2, and IDO pathways, to enhance treatment efficacy. The synergistic effects observed in combination therapies underscore the importance of a multifaceted approach to overcome the complex resistance mechanisms within the TME, paving the way for improved clinical outcomes in CRC.

### 5.5 Dendritic cell vaccine

Dendritic cell (DC) vaccines have emerged as a powerful strategy to counter TME-driven drug resistance in CRC, harnessing the immune system to target tumor cells and their supportive microenvironment. By eliciting robust, antigen-specific immune responses, DC vaccines overcome the limitations of standard therapies, offering a promising pathway to improve treatment outcomes in CRC (Zhang Y. et al., 2024).

One innovative approach integrates DC vaccines with established chemotherapeutic regimens, such as irinotecan combined with infusional 5-fluorouracil and leucovorin (FOLFIRI), to amplify antitumor effects in CRC models. In preclinical studies using the MC38/CEA2 colorectal cancer model, researchers established the maximum tolerated dose of FOLFIRI and subsequently introduced a DC vaccine expressing carcinoembryonic antigen (CEA). This combination significantly enhanced antitumor efficacy compared to FOLFIRI alone, driven by heightened CEA-specific Th1 and cytotoxic T-cell responses. Notably, while FOLFIRI initially reduced tumor burden, it triggered a rebound in immunosuppressive cells, including MDSCs and Tregs, after 14 days. The DC vaccine effectively suppressed this rebound, mitigating the immunosuppressive TME and sustaining antitumor immunity. Mice treated with this combined regimen demonstrated antigen-specific T-cell responses and resistance to tumor rechallenge, highlighting the potential of DC vaccines to reshape the TME and bolster long-term therapeutic success (Kim et al., 2010). Building on this synergy, DC vaccines also target tumor-supportive structures within the TME, such as tumor-derived blood vessels essential for CRC growth and metastasis. In a syngeneic mouse model of CRC, a clinically relevant alpha type-1 polarized DC vaccine (αDC1) was developed to target tumor-derived pericytes expressing DLK1. This αDC1 vaccine induced potent anti-tumor effects by enhancing cytotoxic T lymphocyte activity and disrupting tumor vasculature. By attacking the vascular components of the TME, the αDC1 vaccine not only curtailed tumor progression but also established a foundation for exploring immune-mediated strategies to overcome CRC resistance. This multifaceted approach underscores the versatility of DC vaccines in addressing both cellular and structural elements of the TME, offering a comprehensive strategy against colorectal malignancies (McCormick et al., 2023).

The challenge of targeting CSCs, which fuel tumor recurrence and resistance to conventional therapies, has also been met with innovative DC vaccine-based strategies. In CRC, CSCs expressing aldehyde dehydrogenase 1 (ALDH1) exhibit elevated levels of PD-L1, rendering them resistant to standard treatments. A novel combination immunotherapy, integrating PD-L1-specific chimeric antigen receptor T cells (PD-L1-CAR-T) with CCSC-DC vaccines prepared from CCSC lysates, demonstrated remarkable efficacy. While monotherapy with either PD-L1-CAR-T cells or CCSC-DC vaccines yielded only moderate tumor remission, their combination significantly enhanced tumor cell destruction and alleviated tumor burden in preclinical models. The CCSC-DC vaccine primed T cells to recognize CCSC-specific antigens, synergizing with the targeted cytotoxicity of PD-L1-CAR-T cells. This integrated approach highlights the pivotal role of DC vaccines in sensitizing the immune system to combat resistant CSCs within the TME, offering a transformative strategy for CRC treatment (Liu L. et al., 2021).

These findings underscore the critical role of DC vaccines in overcoming TME-mediated drug resistance in colorectal cancer. By amplifying T-cell responses, suppressing immunosuppressive cells, targeting tumor vasculature, and addressing resistant CSCs, DC vaccines provide a holistic approach to dismantling the barriers posed by the TME. These preclinical insights pave the way for designing advanced chemo-immunotherapeutic and immunotherapeutic strategies, offering hope for improved clinical outcomes in patients with colorectal carcinoma.

## 6 TME-centered classification strategies and implications for personalized therapy in CRC

Given the major role that the TME has been shown to play in mediating therapeutic resistance to CRC, it is of great importance that classification systems of CRC can be developed that take these aspects of the TME into account, and that they can be used in a clinical setting to improve prognostic power and aid in making personalized treatment decisions. Although tumor-intrinsic factors are clearly a driving force of clinical outcomes, the heterogeneity of the TME has proven to be of great importance, and has given rise to TME-based approaches to subtyping that have been developed based on the analysis of bulk transcriptomic data as well as integrating multiple omics layers (Tang et al., 2025). A prime example for a subtyping system is the well-established Consensus Molecular Subtypes (CMS) system, which was originally derived from unsupervised clustering of bulk gene expression datasets from large cohorts of CRC patients. By clustering patients into four major subtypes, CMS reflects major variation in TME composition in the CRC patient population; CMS1 (MSI-immune, ∼14%) is characterized by microsatellite instability, high mutation load, and prominent infiltration by immune cells, especially helper and cytotoxic T cells. These tumors are associated with improved overall survival, but their highly immunogenic nature can lead to T-cell exhaustion, potentially resulting in reduced responses to immunotherapies. CMS2 (canonical, ∼37%) is primarily epithelial in nature, with strong activation of WNT/MYC pathways, low stromal and immune content, and sensitivity to standard chemotherapy. CMS3 (metabolic, ∼13%) is associated with metabolic dysregulation and a moderate immune presence. CMS4 (mesenchymal, ∼23%) has high stromal content, with an abundance of cancer-associated fibroblasts (CAFs), extracellular matrix remodeling, TGF-β activation, angiogenesis, and immunosuppression; these are typically more aggressive, present at more advanced stages, have poorer overall survival, and are associated with high levels of drug resistance (Guinney et al., 2015). This is in line with the principles discussed in this review, where the fibroblast-driven epithelial-to-mesenchymal transition and cytokine-mediated immunosuppression both likely play roles in mediating resistance and are being developed as targets for TME-modifying therapies.

The CMS system has been shown to consistently predict overall survival and the risk of relapse, as well as responses to chemotherapy and targeted agents, and is now being validated as a predictor of immunotherapy response and resistance, with CMS1 tumors being more immunogenic and therefore more likely to respond to immune checkpoint blockade, and CMS4 tumors showing strong stromal barriers that may be overcome using combination approaches, such as TGF-β inhibitors with checkpoint blockade (Dang et al., 2024; Ten Hoorn et al., 2022). This type of approach can be made more feasible for use with clinical samples by the development of bulk-omics methods such as cell deconvolution techniques, which can be used to infer the immune and stromal fractions of the TME in a sample and can be used as the basis to assign it to a CMS category. In turn, this could be used in the context of developing predictive models of response to particular therapies for clinical use in a precision medicine setting. Similar approaches have also been developed in other tumor types based on the TME, which demonstrates the broad utility of unsupervised clustering and machine learning approaches to bulk transcriptomic data to identify clinically relevant subtypes. For instance, unsupervised clustering based on m7G-related features in bladder cancer has been able to identify immune-activated subtypes that have favorable outcomes with immunotherapy (Lai et al., 2022), and immune-related gene expression signatures in adrenocortical carcinoma have been able to classify tumors into inflamed versus immune-excluded TMEs, which can be used for prognostic purposes and to predict responses to checkpoint inhibitors (Lai et al., 2023; Zhang Y. et al., 2023). Such methods, developed in the context of CRC, could therefore be combined with the targets and pathways discussed in the context of therapeutic resistance in this review to further hone the biomarkers of each subtype and prioritize TME-modifying therapies in each setting, as well as better select patient populations that may be suitable for novel combinatorial approaches. It will be crucial to validate the utility of TME-based subtyping systems in clinical trials in order to implement these methods and design effective strategies to overcome therapeutic resistance in CRC.

## 7 Translational and clinical implications of TME components in CRC therapeutic resistance

CRC-associated TME resistance mechanisms are well-established in preclinical models, cell lines, organoids, and xenografts. Translating these into a clinical setting will require high-quality evidence from patient cohorts, validated biomarkers, and druggable therapies. MSI-H tumors, which have an immune-hot TME and are characterized by a high density of infiltrating T cells, are particularly sensitive to ICIs. At 5-year follow-up, phase III KEYNOTE-177 confirmed the superiority of first-line pembrolizumab over chemotherapy in improving overall survival of patients with MSI-H/dMMR mCRC, with most responses durable despite crossover (André et al., 2025a). Similarly, in the prespecified interim analysis of the phase III CheckMate 8HW trial, nivolumab plus ipilimumab showed superior progression-free survival compared with nivolumab alone in MSI-H/dMMR patients treated in the first, second, or third line, supporting the dual ICI approach. In contrast, MSS tumors often show immune-cold and stroma-rich phenotypes leading to ICI primary resistance and low response rates to monotherapy (André et al., 2025b).

For cellular components, high CD8^+^ T-cell density has consistently been shown to be an independent predictor of favorable prognosis in early and advanced CRC and as a predictive biomarker of ICI response in the MSI-H subset, using the international consensus validation of Immunoscore, a digital pathology algorithm that quantifies CD3/CD8+ T cell infiltration to outperform TNM stage in large, multi-center cohorts (Pagès et al., 2018). High NLR, calculated using data from the complete blood count (CBC) test, is an independent prognostic factor for worse outcomes in early and advanced CRC and is associated with chemoresistance in advanced-stage CRC, as identified in several meta-analyses and reviews of multiple patient series (Naszai et al., 2021). In MSS tumors, the abundance of M2-like TAMs and MDSCs is implicated in T cell exclusion and exhaustion, and early phase trials are investigating the combination of CSF-1R inhibitors or anti-IL-6 with ICIs to reprogram the suppressive immune cell population (Xia et al., 2025). NK cell and DC therapy are in preclinical development and show promise in MSI-H animal models, but are largely investigational in clinical studies and likely require combination for MSS indications (Wang Q. et al., 2024).

Among non-cellular components, CAF expressing FAP and stromal deposition of ECM are the most prominent features of the mesenchymal CMS4, associated with high stage and metastatic disease at presentation, and worst overall and relapse-free survival among the CMS subtypes in an international cohort of patients (Guinney et al., 2015). In studies of resected CRC, high stromal FAP expression is associated with aggressive disease progression and poor prognosis (Coto-Llerena et al., 2020). Hypoxia-induced pathways and cytokines (e.g., IL-6 and TGF-β) drive EMT and angiogenesis, with both high FAP expression and serum IL-6 levels identified as negative prognostic markers. FAP-targeting therapies, FAP inhibitor or FAP CAR-T cells, and TGF-β blockers are in early clinical development and have shown to remodel the stromal compartment and allow for increased drug penetration of drugs in MSS models (Wu et al., 2021).

Taken together, evidence from patient-derived data support the use of routine TME profiling such as Immunoscore, NLR, or CMS subtyping for prognostic and treatment decision-making. ICI agents are now approved and improve outcomes of MSI-H, but primary resistance is common in MSS, underscoring the need for combination approaches to overcome suppressive immune cell and stromal barriers. Current clinical trials are investigating macrophage reprogramming, CAF targeting, and hypoxia reversal as potential treatment options for personalization based on biomarker discovery.

Among the cellular and non-cellular TIME components discussed, M2-polarized TAMs, CAFs, and TGF-β signaling emerge as particularly tractable targets for overcoming therapeutic resistance in CRC (Wu H. et al., 2025). M2 TAMs, recruited via CSF1/CSF1R and often polarized by TGF-β or IL-6, drive immunosuppression, chemoresistance, and angiogenesis across multiple modalities. CSF1R inhibitors (e.g., pexidartinib) deplete or reprogram suppressive TAMs, showing preclinical synergy with chemotherapy and ICIs in MSS models. CAFs, marked by high FAP expression, remodel ECM to promote EMT, metastasis, and drug exclusion; FAP-targeted approaches disrupt stromal barriers and enhance effector cell infiltration. TGF-β, secreted by multiple TIME cells including CAFs and TAMs, converges on resistance via EMT induction, T-cell exclusion, and MDSC recruitment, making its blockade a high-leverage intervention. These targets are “tractable” due to selective expression in the TME, availability of clinical-grade agents, and evidence of TIME remodeling in early trials (Gallo et al., 2021; Xun et al., 2020). Recent and ongoing clinical trials underscore progress in TIME-targeted strategies for CRC resistance. Phase I/II trials of CSF1R inhibitors, cabiralizumab or pexidartinib, combined with anti-PD-1 show tolerable safety and TAM depletion in advanced solid tumors (NCT02584647). FAP-targeted therapies, including radioligands and CAR-T, are in early trials for stromal remodeling in MSS CRC, with preliminary responses in refractory cases (NCT04939610). TGF-β inhibitors like vactosertib combined with pembrolizumab demonstrated antitumor activity in metastatic MSS CRC/gastric cancer (Kim et al., 2021), while bintrafusp alfa (TGF-β trap/anti-PD-L1 bifunctional) showed signals in HPV-associated cancers but limited in CRC (Lind et al., 2020). These trials highlight feasibility of TIME reprogramming to sensitize “cold” MSS tumors to ICIs or chemotherapy.

Integrating TIME biomarkers, multi-omic CMS, FAP imaging, or liquid biopsy for MDSCs/TGF-β, into trial design will enable precision selection of patients likely to benefit from macrophage/CAF/TGF-β targeting, potentially converting MSS CRC from resistant to responsive. Combination regimens, CSF1R + PD-1 blockade or FAP + chemotherapy, are poised to address multifactorial resistance, with adaptive trials needed to optimize sequencing and dosing. Ultimately, TIME-directed therapies may shift CRC management toward personalized, resistance-overcoming approaches, improving outcomes beyond MSI-H subsets.

## 8 Conclusion and perspectives

In essence, therapeutic resistance in colorectal cancer arises from modular, interdependent circuits within the tumor microenvironment that integrate immune suppression, metabolic rewiring, stromal barrier formation, and vascular dysfunction. Key shared hubs, TGF-β superfamily signaling, IL-6/STAT3 activation, hypoxic HIF-1α responses, and myeloid-attracting chemokine pathways, orchestrate crosstalk between immune cell populations and non-cellular elements, creating robust barriers to chemotherapy, immunotherapy, and targeted agents. This integrated view shifts the therapeutic paradigm from single-agent or single-target approaches toward multi-modal combinations that simultaneously relieve immune exclusion, reverse exhaustion, normalize metabolism and vasculature, and degrade stromal support. Prioritizing interventions against these convergent nodes, guided by TIME biomarkers, offers a rational path to overcome resistance and achieve durable responses in diverse CRC patient populations.

Despite this progress, significant limitations hinder the translation of TME-focused strategies into clinical practice. Much of the existing data is derived from preclinical models that, although informative, cannot fully mimic the complexity and heterogeneity of the human tumor milieu. The TME evolves dynamically under selective pressure from therapies, yet longitudinal studies capturing these temporal changes remain scarce. Additionally, research often investigates single pathways or cell subsets in isolation, overlooking the synergistic and sometimes redundant networks that sustain resistance. The variability across patients, influenced by genetic background, microbiome composition, and environmental exposures, further complicates the generalization of findings. Finally, the safety, specificity, and feasibility of interventions that target immune and stromal components are not yet well established, raising concerns about off-target effects and potential impairment of host defense mechanisms.

Future perspectives in this field call for a more integrative and systems-level approach. High-dimensional technologies such as single-cell RNA sequencing, spatial transcriptomics, and multiplex imaging can provide unprecedented resolution of cellular interactions within the TME. Patient-derived organoids, xenografts, and advanced co-culture systems offer promising platforms to evaluate the dynamic interplay between tumor and microenvironment under therapeutic pressure. These tools will allow the identification of actionable vulnerabilities and context-dependent biomarkers that predict resistance or sensitivity. Moreover, comprehensive mapping of TME evolution across treatment timelines will be essential to design adaptive therapeutic strategies that preempt or reverse resistance mechanisms as they arise. From a therapeutic standpoint, rational combination regimens that simultaneously target malignant cells and their supportive niches are particularly promising. Strategies such as dual checkpoint blockade with modulation of immunosuppressive cytokines, inhibition of pro-tumor stromal pathways, or reprogramming of macrophages and fibroblasts into tumor-restraining phenotypes could redefine the efficacy of current treatments. Advances in nanotechnology and drug delivery systems may further enhance the precision and safety of TME-targeting interventions, reducing systemic toxicity. Importantly, integrating TME-based biomarkers into clinical trial design can improve patient stratification, ensuring that therapies are directed toward individuals most likely to benefit. Such personalized strategies are critical for addressing the variability in treatment response observed among CRC patients. Even more, single-cell technologies surpass bulk-omics by unveiling TME heterogeneity and therapeutic resistance mechanisms in unprecedented detail. scRNA-seq has pinpointed key immune subsets driving ICI responses in solid tumors. Extending these approaches to “cold” MSS CRC promises to reveal resistance networks and personalized TME strategies.

In conclusion, the TME represents both a formidable barrier and a fertile therapeutic opportunity in the management of colorectal cancer. Deciphering its multifaceted roles in drug resistance provides a blueprint for designing next-generation therapies that transcend conventional tumor-centric approaches. Although challenges remain, ranging from the complexity of cellular interactions to the hurdles of clinical translation, emerging technologies and interdisciplinary collaborations hold great promise. By bridging mechanistic insights with precision medicine, the strategic targeting of the TME may ultimately overcome therapeutic resistance, improve long-term outcomes, and bring us closer to durable cures for patients with colorectal cancer.
